# Exploration of Overlooked *Aduncodinium*‐Like (Pfiesteriaceae, Dinophyceae) Diversity

**DOI:** 10.1111/jeu.70116

**Published:** 2026-08-21

**Authors:** Albert Reñé, Aika Yamaguchi, Takeo Horiguchi, Kunihiko Watanabe, Mona Hoppenrath

**Affiliations:** ^1^ Dpt. Biologia Marina i Oceanografia Institut de Ciències del Mar (CSIC) Barcelona Catalonia Spain; ^2^ Department of Biological Sciences, Faculty of Science Hokkaido University Sapporo Japan; ^3^ Senckenberg am Meer German Centre for Marine Biodiversity Research (DZMB) Wilhelmshaven Germany

**Keywords:** benthic dinoflagellates, *Katodinium*, microscopy, morphology, rDNA, sand‐dwelling, taxonomy

## Abstract

The dinoflagellate genus *Aduncodinium* was erected to encompass *Katodinium glandula*, belonging to the family Pfiesteriaceae. Other described species, like *K. asymmetricum* , show *Aduncodinium*‐like characters and probably belong to this genus. These two species are commonly reported in the literature. However, detailed morphological characterization of observed specimens is rarely provided mostly due to difficulties in culturing and studying these thin‐walled dinoflagellates. Likewise, molecular information for *Aduncodinium*‐like species is scarce. In this study, we provide a morpho‐molecular characterization of *Aduncodinium*‐like morphospecies mostly detected in coastal sediments from Spain, Germany, Japan, and Canada. The information collected and the detections reported in the literature, mostly referring to *K. glandula* or *K. asymmetricum* , highlight the existence of a largely overlooked diversity of *Aduncodinium*‐like taxa, and the existence of cryptic diversity in some morphospecies. In fact, up to 16 different morphospecies are reported in this study, some of them supported by their molecular information, which can be assigned to different morphogroups. The information collected results in the description of the new species *Aduncodinium robustum* sp. nov.

## 1 Introduction

The family Pfiesteriaceae comprises small heterotrophic dinoflagellate species with a thin theca and a plate formula: APC (Po, X), 4–5′, 0–2a, 5–7″, 6c, 6+s (pc + 5–?s), 5‴, 0p, 2⁗. Pfiesteriaceans received special attention during the 1990s because *Pfiesteria piscicida* was pointed out as the causative agent of fish lesions and mortality, and human illness occurring in the eastern coast of United States (Steidinger et al. 1996). *Pfiesteria* and *Pfiesteria*‐like relatives are voracious predators, not only affecting fish, but also actively feeding on other microalgae, including dinoflagellates (Burkholder and Glasgow 1997; Lim et al. 2014). Those studies determined the presence of many unknown related *Pfiesteria*‐like dinoflagellates, leading to the description of many species belonging to new genera (Burkholder and Glasgow 1997).

The family Pfiesteriaceae is widely recognized in the literature. However, the established systematics of Peridiniales and the family Thoracosphaeraceae was re‐organized, recognizing three robust clades (Elbrächter et al. 2008; Gottschling et al. 2012). The family Ensiculiferaceae was first separated (Li et al. 2020), followed by Calciodinelloideae and Thoracosphaeroideae (Kretschmann et al. 2022). The latter encompasses the calcareous genus *Thoracosphaera*, a clade including heterotroph pfiesteriacean species and other autotrophic relatives like *Apocalathium*, *Chimonodinium* and *Fusiperidinium* (Craveiro et al. 2017, 2011; McCarthy et al. 2018). In this study, we will refer to the family Pfiesteriaceae to ensure consistency with previous references to this family. The family currently includes the phagotrophic dinoflagellate genera *Pfiesteria*, *Cryptoperidiniopsis*, *Luciella*, *Stoeckeria*, *Tyrannodinium* and *Aduncodinium* (Calado et al. 2009; Jeong et al. 2005; Kang et al. 2015; Mason et al. 2007; Steidinger et al. 2006, 1996), as well as the parasitic genera *Amyloodinium* and *Paulsenella* (Kühn and Medlin 2005). The genus *Pseudopfiesteria* was erected to encompass *Pfiesteria shumwayae* (Litaker et al. 2005), but this taxonomic transfer is under debate (Marshall et al. 2006). Some *Katodinium* representatives have been recently proved to belong to Pfiesteriaceae. Consequently, the genus *Aduncodinium* was erected to accommodate *Katodinium glandula* (Kang et al. 2015; Reñé et al. 2025), and the genus *Speroidium* was erected to encompass *Katodinium fungiforme* (Moestrup and Calado 2018). Most Pfiesteriaceae representatives inhabit brackish/marine waters, even though *Tyrannodinium edax* is common in freshwater ponds and lakes (Calado 2011; Calado et al. 2009), and *Speroidium* representatives and *P. shumwayae* can be found in freshwater environments (Moestrup and Calado 2018). Pfiesteriaceans inhabit both the planktonic and benthic compartment, and hidden diversity was discovered from resting cysts obtained in sediments from Chinese waters (Tao et al. 2024).

The genus *Katodinium* Fott encompasses species having a remarkably large epicone compared to the hypocone, but many of them show no resemblance to the type species *K. nieuportense* (W.Conrad) Fott (Calado 2011). *Katodinium* species have been successively transferred to other genera and nowadays only 15 species remain affiliated to the genus out of 56 species described, according to AlgaeBase (Guiry and Guiry 2025; Table S1). In the case of marine species, most reports in the literature refer exclusively to *K. glandula* and *K. asymmetricum* , and many of them correspond to observations from benthic habitats. As previously stated, *K. glandula* is a pfiesteriacean and it has been transferred to the genus *Aduncodinium* (Kang et al. 2015; Reñé et al. 2025). Regarding *Katodinium asymmetricum* , it was originally described as *Gymnodinium asymmetricum* (Massart 1920), later on referred to *Massartia* as “*Massartia asymetrica*” (Biecheler 1952), and finally transferred to the genus *Katodinium* (Fott 1957; Loeblich III 1965). Even though it shows *Aduncodinium*‐like characters, *K. asymmetricum* still remains affiliated to the genus *Katodinium*.

A large diversity of *Aduncodinium*‐like species, detected in coastal benthic and planktonic habitats, is recognized in the images provided in the literature (e.g., Al‐Qassab et al. 2002; Gómez and Artigas 2014; Hulburt 1957; Larsen 1985), but despite efforts conducted to expand the knowledge on pfiesteriaceans, such *Aduncodinium*‐like diversity remains poorly characterized. Consequently, the objective of this study is to explore the diversity of *Aduncodinium*‐like taxa based on information gathered from observations conducted worldwide from benthic habitats, providing their morphological characteristics and molecular information. Based on this information, a new species *Aduncodinium robustum* sp. nov. is described. A thorough documentation of *Aduncodinium*‐like taxa reports from the literature is included to determine the extent of overlooked diversity of species belonging to this genus.

## 2 Materials and Methods

### 2.1 Sampling

Details on the sampling procedures conducted in Catalonia, Spain (NW Mediterranean Sea) have been previously published (Reñé et al. 2021). Briefly, sublittoral sandy sediments were obtained in Barceloneta Beach (41°23′04.5″ N, 2°11′55.7″ E), L'Estartit (42°03′05.3″ N, 3°12′07.8″ E) and Castelldefels (41°15′35″ N, 1°55′51″ E) from April to September 2017 and from sporadic sampling conducted during summer months along 2018–2023. Sediment cores were obtained at 2 m depth. Microorganisms from the first 5 cm were eluted using the seawater‐ice method (Uhlig 1964) and observed under light microscopy. In Germany (at Sylt and Wilhelmshaven) and Canada (at Boundary Bay, Brady's Beach and Pachena Beach, British Columbia) eulittoral sandy sediments were obtained during low tide and cells were also extracted with the seawater‐ice method as has been published (Hoppenrath 2000a; Hoppenrath et al. 2020; Hoppenrath and Leander 2007; Reñé et al. 2024). The sand samples from Japan were collected in Ishikari Beach, Hokkaido and the details on the sampling site and procedures have been published in Hoppenrath et al. (2007) and Watanabe et al. (2014).

### 2.2 Light and Scanning Electron Microscopy

Living and fixed samples from the Catalan coast were observed and cellular measurements were conducted under a phase‐contrast Leica DM‐IRB inverted microscope (Leica Microsystems, Wetzlar, Germany), equipped with an epifluorescence filter set, and connected to a ProgRes C10 (Jenoptik Laser, Optik Systeme GmbH, Jena, Germany) digital camera. Epifluorescence microscopic examination of the thecal plate tabulation was performed on fixed cells stained with two to three drops of Calcofluor white at a final concentration of 10–20 μg mL^−1^ (Fritz and Triemer 1985). Differential interference contrast (DIC) images were obtained by a Nikon Eclipse Ts2R‐FL inverted microscope. For scanning electron microscopy (SEM) observations, Lugol‐fixed subsamples were filtered into a 3.0–5.0 μm polycarbonate filter, and washed in seawater and distilled water for 15 min. A subsequent dehydration was carried out in a 25%, 50%, 75%, 90%, 96%, and 100% ethanol series for ca. 10 min. The final step of 100% ethanol was repeated twice. The filters were critical‐point dried with liquid carbon dioxide in a BAL‐TEC CPD030 unit (Leica Mycrosystems, Austria). The dried filters were then mounted on stubs, sputter coated with gold–palladium and examined with a HITACHI S‐3500 N scanning electron microscope (Hitachi High Technologies Corp., Tokyo, Japan) at the Electron Microscopy Facility (ICM‐CSIC). Some samples were observed in a Tescan VEGA3 microscope (Elektronen‐Optik‐Service GmbH, Dortmund, Germany) at 15 kV, following the procedures described in Reñé and Hoppenrath (2019).

In Germany, cells were identified with a Leitz Fluovert FS inverted microscope and further examined with a Leica DMRB microscope. For SEM, specimens were fixed in Lugol's solution, filter‐mounted, rinsed with distilled water, and subsequently dehydrated with 30% ethanol and with dimethoxypropane (Merck). The filter was air‐dried and then sputter‐coated with gold–palladium (Bal‐Tec SCD 050) and examined with a Zeiss DSM 940A scanning electron microscope (Carl Zeiss, Oberkochen, Germany) (Hoppenrath 2000b).

In Canada, cells were observed with a Leica DMIL inverted microscope (Wetzlar, Germany) connected to a PixeLink Megapixel color digital camera (Hoppenrath et al. 2020; Hoppenrath and Leander 2007). For SEM, samples were fixed with Lugol's solution, transferred onto a 5 μm polycarbonate membrane filter (Corning Separations Div., Acton, MA, USA), washed with distilled water, dehydrated with a graded series of ethanol, rinsed twice in hexamethyldisilazane (HMDS), and oven‐dried at 65°C. Filters were mounted on stubs, sputter‐coated with gold, and viewed under a Hitachi S4700 scanning electron microscope (Hoppenrath et al. 2020).

Living cells from Japan were isolated and observed using an OLYMPUS BX50 microscope equipped with Nomarski interference optics (OLYMPUS, Tokyo, Japan). Photographs were taken using a Nikon digital camera DS‐Fi1 (Nikon, Tokyo, Japan). After taking photographs, the coverslip was removed and the observed cell was used for DNA extraction (Takano and Horiguchi 2004). For SEM, the isolated cell was put on a small glass plate coated with poly‐L‐lysine. The attached cell was fixed in Lugol's iodide overnight, rinsed a few times in distilled water, and dehydrated gradually through an ethanol series (30%, 50%, 70%, 90%, 95%, and 100%). The fixed cell was critical‐point dried, sputter‐coated with gold, and observed with a Hitachi S‐3000 N scanning electron microscope.

### 2.3 PCR Amplification and Sequencing

Specimens of interest observed by light microscopy were manually isolated using a glass capillary micropipette and frozen until processed. Single cell isolates A37 and A38 were obtained from Castelldefels beach on 24th July 2017. They were subjected to a first 25 μL PCR using EK‐82F–28S‐1611R primers with a PCR mixture containing 2.5 μL of 10× buffer (TaKaRa Bio), 1.5 mM MgCl_2_, 1 U of TaKaRa Taq DNA polymerase (TaKaRa Bio), 0.2 mM of each dNTP, and 0.4 mM of each primer. PCR conditions were as follows: initial denaturation for 3 min at 95°C, followed by 6 cycles of 15 s at 95°C, 30 s at 58°C–53°C, decreasing 1°C each cycle, and 2 min at 72°C, and 34 additional cycles at annealing temperature of 52°C, followed by a final extension step for 5 min at 72°C. The resulting product was used as template for semi‐nested PCRs to amplify the nuclear SSU and LSU rDNA regions independently, using primers EK‐82F–EK‐1520R, and 28S‐1F–28S‐803R, respectively. A subsequent semi‐nested PCR was conducted to amplify the nuclear SSU rDNA fragment using the primers DIN464F–EK‐1520R. The PCR mixture contained 1 μL of template, 2.5 μL of 10× buffer (Invitrogen, Thermo Fisher Scientific Corp.) containing 15 mM MgCl_2_, 1.25 U of Platinum Taq DNA polymerase (Invitrogen, Thermo Fisher Scientific Corp.), 0.2 mM of each dNTP, and 0.4 mM of each primer. PCR conditions were as follows: initial denaturation for 2 min at 94°C, 35 cycles of 15 s at 94°C, 30 s at 55°C, and 1 min at 72°C, followed by a final extension step for 5 min at 72°C. Four micro liter of PCR products were electrophoresed in an agarose gel and then visualized under UV illumination. PCR products were purified using ExoSAP‐IT (Applied Biosystems, Thermo Fisher Scientific Corp.). The single‐cell isolate SC535, isolated at L'Estartit beach on 25th June 2015, was subjected to several rounds of freezing/thawing, and a single‐cell 50 μL PCR was conducted with a PCR mixture containing 5 μL of 10× buffer (Qiagen), 1.25 U of Taq DNA polymerase (Qiagen), 0.2 mM of each dNTP, and 0.8 mM of the primers D1R and D3B (Scholin et al. 1994) for the amplification of the partial nuclear LSU rDNA region. The PCR conditions were as follows: initial denaturation for 5 min at 95°C, 40 cycles of 20 s at 95°C, 30 s at 55°C, and 1 min at 72°C, followed by a final extension step for 7 min at 72°C. Four micro liter of the PCR products were electrophoresed for 20–30 min at 120 V in a 1.2% agarose gel and then visualized under UV illumination.

Canadian specimens isolated from a raw sample were washed with sterile filtered seawater and 5 (*Aduncodinium* cf. *asymmetricum*) or 7 (for *Aduncodinium* spec 4) cells deposited in a 1.5 mL Eppendorf tube (Dia‐Med Lab Supplies Inc., Mississauga, ON, Canada). Genomic DNA was extracted using the MasterPure Complete DNA and RNA Purification Kit (Epicenter, Madison, WI, USA), final step with 25 μL distilled water. DNA amplification was carried out using 24 μL of this extract and puReTaq Ready‐To‐Go PCR Beads (Amersham Biosciences, Piscataway, NJ, USA). The protocol using universal eukaryotic primers (PF1‐R4) was described in (Hoppenrath and Leander 2007, 2010). PCR products corresponding to the expected size were gel isolated and cloned into the pCR2.1 using the TOPO TA cloning kit (Invitrogen, Carlsbad, CA, USA). One clone from both taxa was sequenced with ABI BigDye reaction mix (Applied Biosystems, Foster City, CA, USA) using the vector primers and internal primers oriented in both directions.

The isolated cell from Japan was photographed and transferred to 10 μL of Quick Extract FFPE DNA Extraction Solution (Epicenter, Madison, WI, USA) and incubated at 56°C for 1 h, then 98°C for 2 min. The resulting solution was used as DNA template for subsequent PCR amplification. Almost the entire SSU rRNA gene and a partial length of LSU rRNA gene were amplified using the primer sets SR1kuni‐SR12b and D1‐R2 (Watanabe et al. 2014). For the second PCR, 0.5 μL of the first PCR product was used as DNA template, and five pairs of primers were used for nuclear SSU rDNA amplification and two pairs of primers were applied for nuclear LSU rDNA amplification (Watanabe et al. 2014). PCR conditions consist of one initial denaturation stage at 93°C for 5 min, followed by 35 cycles of denaturation at 93°C for 30 s, annealing at 55°C for 30 s, and extension at 72°C for 30 s; and final extension cycle at 72°C for 7 min. The PCR products were sequenced directly using the ABI PRISM BigDye Terminator Cycle Sequencing Kit (Perkin‐Elmer Applied Biosystems, Foster City, CA, USA) and a DNA autosequencer ABI PRISM310 Genetic Analyzer (Perkin‐Elmer Applied Biosystems, Foster City, CA, USA). For the remaining locations, the Sanger sequencing of each fragment was conducted by external services using forward and reverse primers and an ABI 3730XL DNA analyzer (Germany: Macrogen, Netherlands; Spain: Genoscreen, France; Canada: University of British Columbia NAPS Unit). All NCBI accession numbers of sequences generated are provided in Table 1. Information related to primers used to amplify the target regions is provided in Table S2.

**TABLE 1:** Information about the observed species diversity, including their cell size, plate tabulation, location of detection, and NCBI accession numbers.

Taxon	Length [μm]	Width [μm]	Tabulation	Location	18S rDNA	28S rDNA
*A. robustum*	24–40	21–30	APC 4′ 2a 6″ 5–6?c 2+?s 6‴ 2⁗	Castelldefels, Catalonia, Spain	PZ377045; PZ377046	PZ399186; PZ399187
*A*. cf. *robustum*	21–31	19–28	APC 4′ 2a 6″ ?c1+?s 5‴ 2⁗	Pachena Beach, BC, Canada		
*A. glandula*	12–24		APC 4′ 2a 6″ 6c 4s 5‴ 2⁗	Suma Beach, Kobe, Japan	PV292301^a^	PV292301^a^
*A. glandula*	10–29	8–25	APC 4′ 2a 6″ 6c 4+s 5‴ 2⁗	Masan Bay, Korea	LK934662^b^	LK934662^b^
*A*. cf. *glandula*	20–34	15–29	APC 4′ 2a 6″ ?c ?s 6‴ 2⁗	Sylt, Germany		
*A*. cf. *asymmetricum*	14–20	13–16	APC 4′ 1a 6″ ?c ?s 5‴ 2⁗	Ishikari Beach, Hokkaido, Japan	PZ377049	PZ399189
*A*. cf. *asymmetricum* elongated	20–22	12–14	APC 4′ 2a 6″ ?c ?s 5?‴ 2?⁗	Barcelona, Catalonia, Spain		
*A*. cf. *asymmetricum* elongated	13–21	8–13	APC 4′ 2a 6″ ?c ?s 5‴ 2⁗	Sylt, Germany		
*A*. cf. *asymmetricum* rounded	—	—	APC 4′ 1?a 6″ ?c ?s ?‴ ?⁗	Sylt, Germany		
*A*. cf. *asymmetricum* rounded	15	12	n.d.	Wilhelmshaven, Germany	ON015058^c^	ON015071^c^
*A*. cf. *asymmetricum* elongated	14–17	10–11	APC 4′ 1a 6″ ?c ?s 5?‴ 2?⁗	Boundary Bay, BC, Canada		
*A*. cf. *asymmetricum* rounded	17	14	APC 4′ 2?a 6?″ ?c ?s ?‴ ?⁗	Pachena Beach, BC, Canada		
*A*. spec 1	30–37	21–27	n.d.	Barcelona, Catalonia, Spain		
*A*. spec 2	23–34	16–20	APC 4′ 2?a 6?″ ?c ?s 5?‴ ?⁗	Sylt, Germany		
*A*. spec 3	19–23	13–15	APC 4′ 1a 6?″ ?c ?s 5?‴ ?⁗	Boundary Bay, BC, Canada		
*A*. spec 4	27–40	17–20	n.d.	Pachena Beach, BC, Canada	PZ377048	
*A*. spec 5	19–24	15–17	APC 4′ 2a 6″ ?c ?s 5–6‴ 2⁗	L'Estartit, Catalonia, Spain		
*A*. cf. *asymmetricum*	n.d.	n.d.	n.d.	Brady's Beach, BC, Canada	PZ377047	
*A*. cf. *asymmetricum* rounded	—	—	n.d.	Wilhelmshaven, Germany	PV292344^a^	
*Aduncodinium*‐like sp.	n.d.	n.d.	n.d.	L'Estartit, Catalonia, Spain		PZ399188

^a^ Reñé et al. (2025).

^b^ Kang et al. (2015).

^c^ Reñé et al. (2024).

### 2.4 Phylogenetic Analyses

A phylogenetic reconstruction was performed for nuclear SSU and LSU rDNA sequences, including the *Aduncodinium*‐like sequences generated in this study, representatives of the family Pfiesteriaceae, members of the family Calciodinellaceae (= Scrippsiellaceae), and *Heterocapsa* sequences as outgroup. The sequences collected were aligned using the online version of MAFFT v.7 under the “auto” option (Katoh et al. 2019) and the alignments were then manually curated. The final alignment for the nuclear SSU rDNA phylogeny included 106 sequences and 1787 positions, and the final alignment for the nuclear LSU rDNA included 113 sequences and 941 positions.

A maximum likelihood (ML) phylogenetic tree was constructed with RAxML v8.0 (Stamatakis 2014), inferring the best tree from 1000 different starting trees and using GTR + GAMMA as model. All free model parameters were estimated by RAxML. Bootstrap statistical support was evaluated using 1000 pseudoreplicates.

## 3 Results

### 3.1 Molecular Diversity of *Aduncodinium* Representatives

**FIGURE 1:**
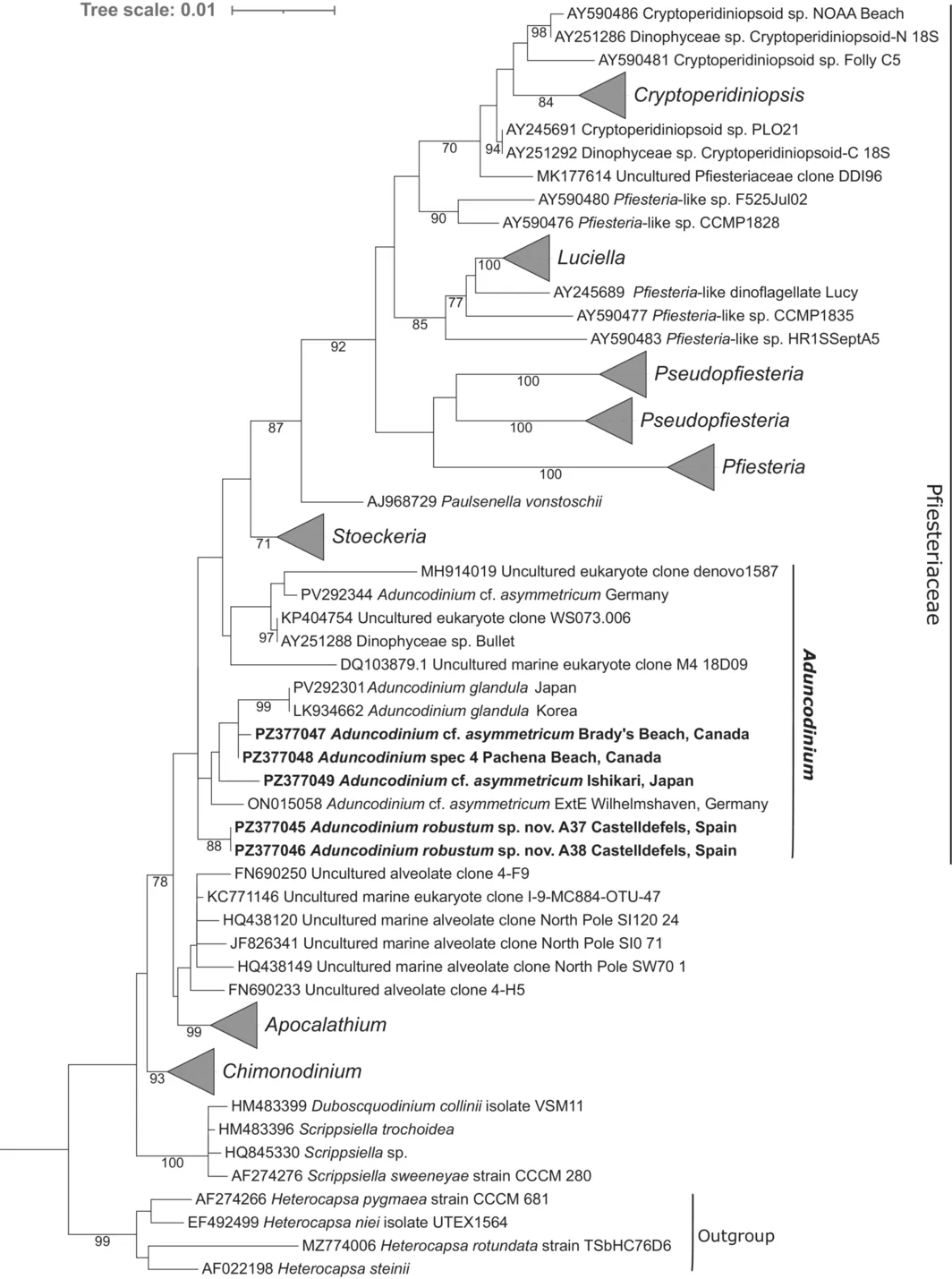
Phylogenetic tree of SSU rDNA sequences covering the diversity of Pfiesteriaceae representatives. Calciodinellaceae (= Scrippsiellaceae) representatives are used as outgroup. Sequences in bold were obtained in this study. Numbers in the nodes represent statistical support (bootstrap values). Only values > 70% are shown.

**FIGURE 2:**
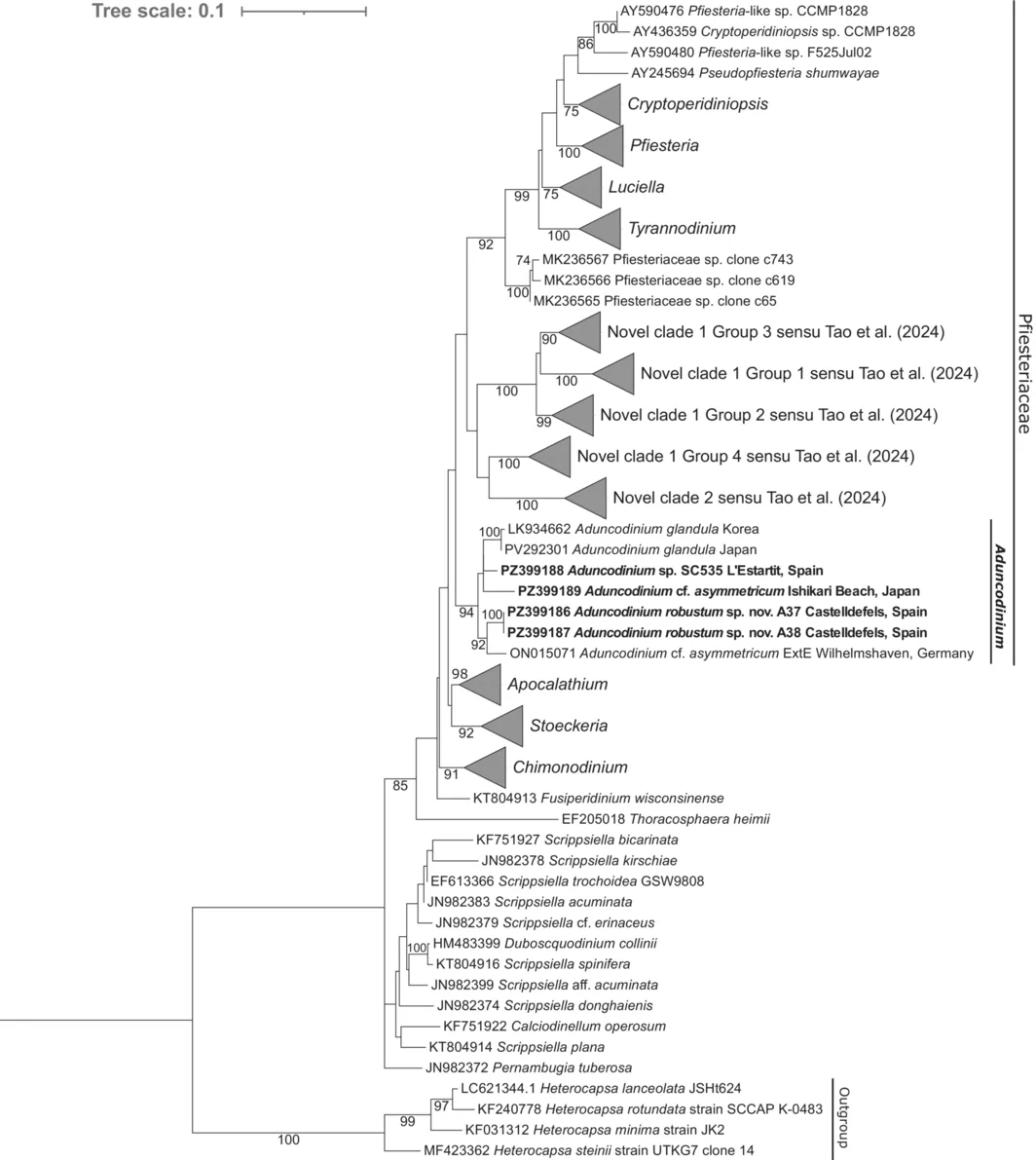
Phylogenetic tree of LSU rDNA sequences covering the diversity of Pfiesteriaceae representatives. Calciodinellaceae (= Scrippsiellaceae) representatives are used as outgroup. Sequences in bold were obtained in this study. Numbers in the nodes represent statistical support (bootstrap values). Only values > 70% are shown.

The sequences of the *Aduncodinium*‐like taxa did not group in a single clade and statistical supports were low, based on SSU rDNA phylogeny (Figure 1). However, they formed a monophyletic clade with high statistical support (94% bootstrap) in the LSU rDNA phylogeny (Figure 2). In the SSU rDNA phylogeny, *Aduncodinium*‐like sequences were distributed among several paraphyletic lineages preceding the clades containing the other Pfiesteriaceae genera, that is, *Cryptoperidiniopsis*, *Luciella*, *Pseudopfiesteria*, *Pfiesteria*, *Paulsenella* and *Stoeckeria*. A first “*Aduncodinium*” clade was found, including the sequences of *A. robustum* sp. nov. A second clade included the sequences of *A*. cf. *asymmetricum* (ExtE, Wilhelmshaven) from Germany, *A*. cf. *asymmetricum* (Ishikari) from Japan, *A. glandula* from Korea and Japan, *A*. cf. *asymmetricum* (clone 6, Bamfield, Brady's Beach, Vancouver Island, BC) from Canada, and *Aduncodinium* spec 4 (clone 1, Pachena Beach, Vancouver Island, BC) from Canada. However, this clade was not supported statistically. Finally, the sequences of remaining *Aduncodinium*‐like members, comprising *Aduncodinium* cf. *asymmetricum* (Wilhelmshaven) from Germany, the “Bullet” strain and two environmental sequences formed a sister clade but again showing no statistical support.

In LSU rDNA phylogeny (Figure 2), the phylogenetic position of *Aduncodinium*‐like sequences was consistent with that inferred from the SSU rDNA gene. The only noticeable difference was that *Stoeckeria* sequences did not group with the other Pfiesteriaceae genera, but outside of this clade, clustering with *Apocalathium* sequences. In this case, *Aduncodinium*‐like sequences formed a single clade with high statistical support (94% bootstrap), including the sequences of *Aduncodinium glandula* from Korea and Japan, *A*. cf. *asymmetricum* (Ishikari) from Japan, *A. robustum* sp. nov. from Spain, *A*. cf. *asymmetricum* ExtE from Germany and *Aduncodinium* sp. SC535 from Spain. The novel environmental clades found by Tao et al. (2024) were recovered, but they showed no direct relationship with *Aduncodinium*/“*Katodinium*” sequences. Unfortunately, LSU rDNA sequences belonging to the “Bullet” strain and *Aduncodinium* cf. *asymmetricum* Germany, which formed a sister group with the previous sequences in the SSU rDNA phylogeny, are not available.

### 3.2 Morphological Diversity of *Aduncodinium* Representatives

The morphological information collected from specimens observed at the studied locations resulted in the documentation of 16 different morphospecies (Table 2). A description of the morphological features observed for the different morphospecies is provided, including the formal description of a new species. In all cases, specimens are heterotrophic, showing no pigmentation, but colorless granules and yellow‐green to orange, red or brown ingestion bodies, sometimes reaching large dimensions, are commonly observed in the episome. Even though a large morphological and molecular diversity was observed, some of the morphospecies documented at the different studied locations could be conspecific. However, we refrain from suggesting it until all of them are completely characterized.

**TABLE 2:** Morphological comparison of the observed species diversity.

Taxon	Anterior intercalary plates	Postcingular plates	Cingulum	Precingular list	1″ list extension	Sd list	Left sulcal list	Pore size classes	Ornamentation
*A. robustum* (Castelldefels, Spain)	2a connected	6‴	V‐shaped	Yes	Yes	Posterior pointing	Yes (1‴ and 1⁗) extension from 1⁗	One	Smooth
*A*. cf. *robustum* (Pachena Beach, Canada)	2a connected	5‴	V‐shaped	Yes	No	Posterior pointing	Yes? (1‴)	One	Smooth
*A. glandula* (Korea and Japan)	2a connected	5‴	Straight	No	No	Left list with no extension	No	One	Smooth
*A*. cf. *glandula* (Sylt, Germany)	2a connected	6‴	Straight	No	No	n.d.	Yes (1‴)	One	Smooth
*A*. cf. *asymmetricum* (Ishikari, Japan)	1a	5‴	Straight	?	No	n.d.	Yes (1‴)	One?	Tightly dotted/pusticulated
*A*. cf. *asymmetricum* (elongated, Barcelona, Spain)	2a no contact	5?‴	Reverse V‐shaped	Yes?	No	n.d.	Yes (1⁗)	One?	Smooth
*A*. cf. *asymmetricum* (elongated, Sylt, Germany)	2a no contact	5‴	Reverse V‐shaped	Yes?	No	n.d.	Yes (1⁗)	One	Smooth
*A*. cf. *asymmetricum* (rounded, Sylt, Germany)	n.d.	n.d.	Straight or slightly reverse V‐shaped	No?	No	n.d.	No	One?	Smooth
*A*. cf. *asymmetricum* (rounded, Wilhelmshaven, Germany)	n.d.	n.d.	Straight	n.d.	No	n.d.	n.d.	n.d.	n.d.
*A*. cf. *asymmetricum* (elongated, Boundary Bay, Canada)	1?a	4–5?‴	Reverse V‐shaped	Yes?	Yes, tiny	n.d.	Yes (1⁗) + right sulcal list	One	Smooth
*A*. cf. *asymmetricum* (rounded, Pachena Beach, Canada)	2?a	n.d.	n.d.	Yes?	n.d.	n.d.	n.d.	One	Characteristic delicate ridges
*A*. spec 1 (Barcelona, Spain)	n.d.	n.d.	Straight	n.d.	n.d.	n.d.	Yes (1⁗)?	n.d.	n.d.
*A*. spec 2 (Sylt, Germany)	2?a	n.d.	Straight	No	No	n.d.	n.d.	One	Smooth
*A*. spec 3 (Boundary Bay, Canada)	1a	5–6	Straight?	n.d.	n.d.	n.d.	n.d.	Two	Smooth
*A*. spec 4 (Pachena Beach, Canada)	n.d.	n.d.	Straight	No	No	n.d.	No?	One	Smooth
*A*. spec 5 (L'Estartit, Spain)	2a connected	5–6‴	Straight	No	No	Posterior pointing + rounded	Yes (1⁗) + wing in 6‴	One	Smooth

#### 3.2.1 *Aduncodinium robustum* sp. nov. Reñé & Hoppenrath (Figures 3 and 4)

**FIGURE 3:**
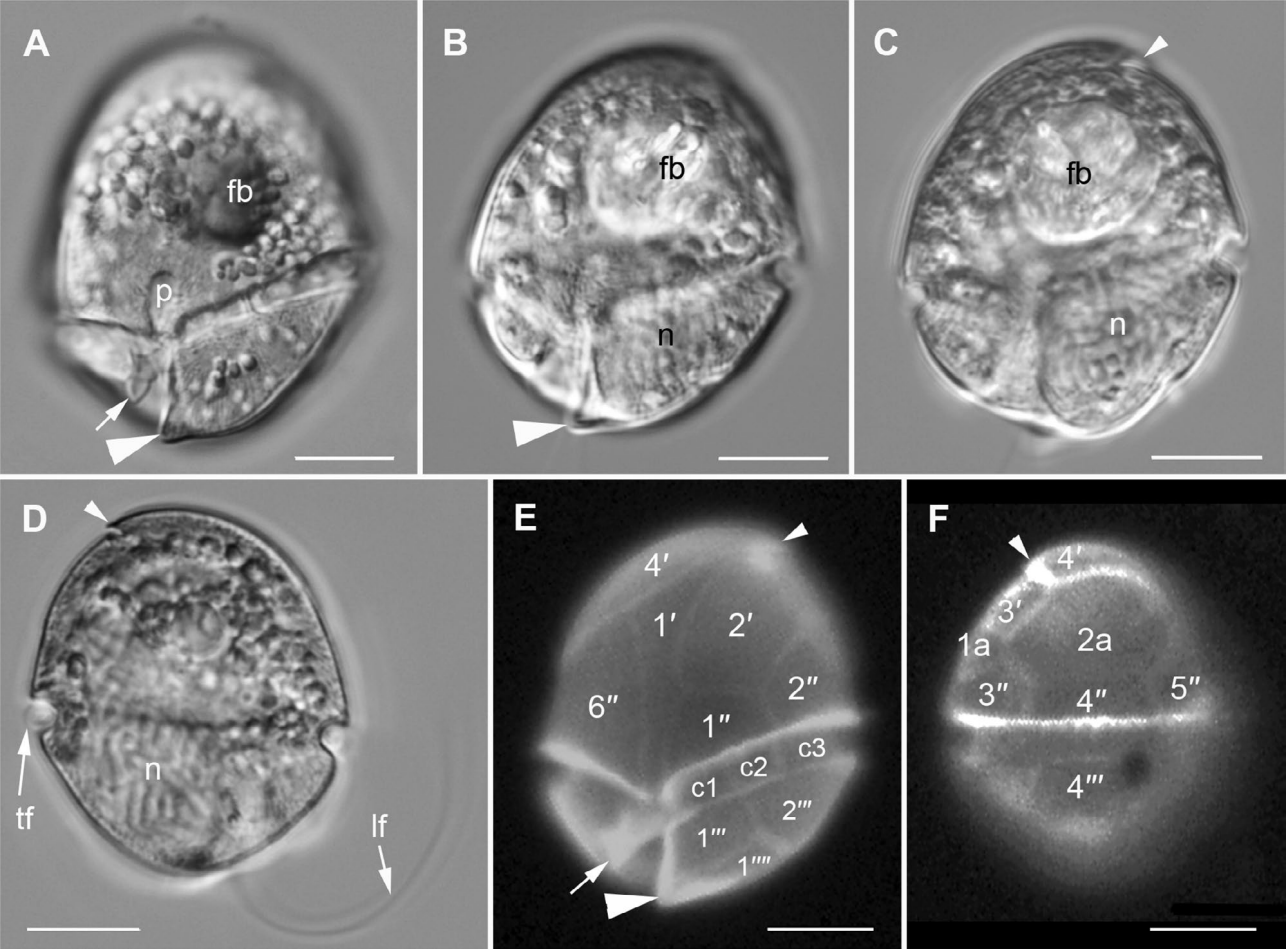
Light microscopy images of *Aduncodinium robustum* sp. nov. obtained by differential interference contrast (A–D) and epifluorescence (E, F). (A–C) Ventral view of the cell. (D) Dorsal view of the cell. (E, F) Epifluorescence ventral and dorsal view of the cell, respectively, showing the thecal plate pattern. The arrowheads indicate the apical hook in (B, D), and the list of 1⁗ plate in (A–C). The arrow indicates the list of the sulcal plate in (C and E). fb, food body; lf, longitudinal flagellum; *n*, nucleus; *p*, pusule; tf, transverse flagellum. Scale bars = 10 μm.

**FIGURE 4:**
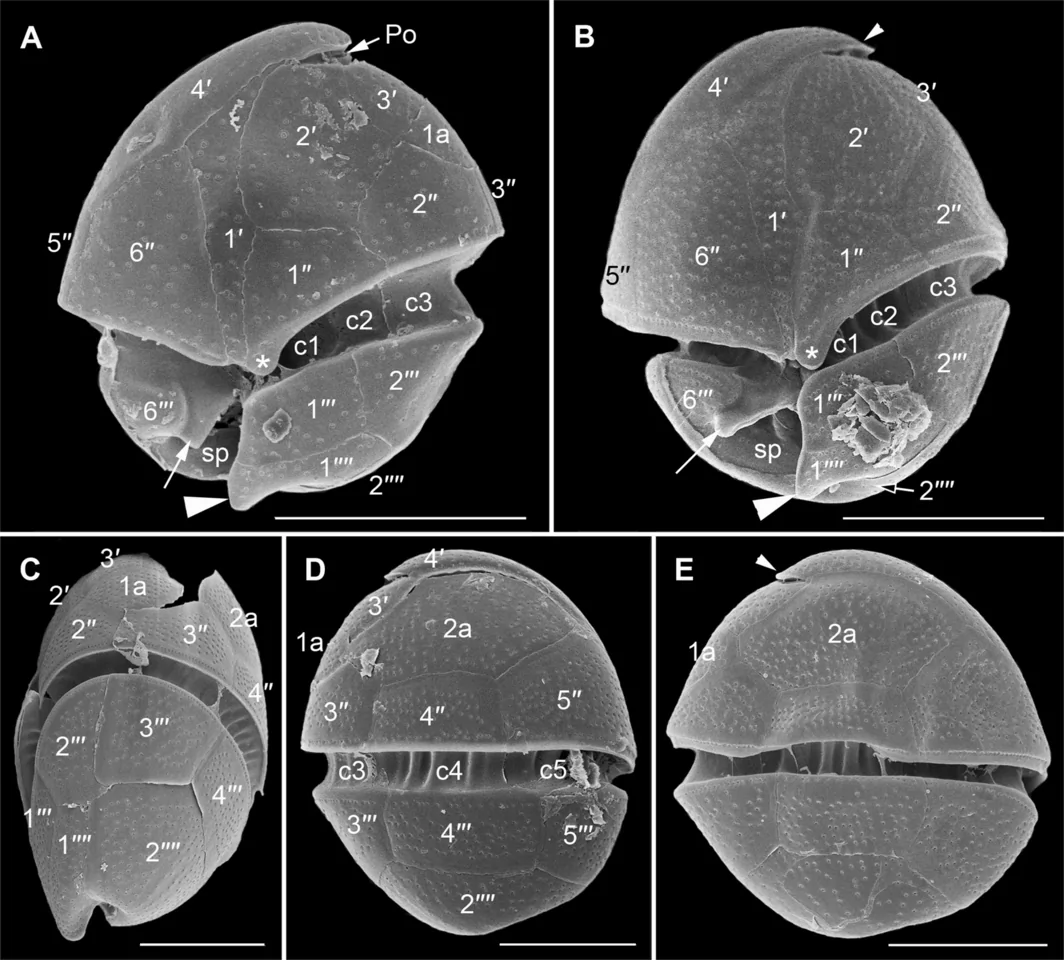
Scanning electron microscopy images showing the plate pattern of *Aduncodinium robustum* sp. nov. (A, B) Ventral view. (C) Left lateral view of the hyposome. (D, E) Dorsal view. The small arrowheads indicate the apical hook in (B, E): large arrowheads indicate the list of 1″ plate, and arrows indicate the list of the sulcal plate in (A and B). Scale bars = 10 μm.

Description: Thecate, heterotrophic dinoflagellate with obliquely dorsoventrally flattened cells. Episome larger than hyposome. Tabulation (Kofoid system) APC, 4′, 2a, 6″, 5–6?c, 2+?s, 6‴, 0p 2⁗. Cingulum postmedian slightly descending, less than one cingular width, forming a V‐shape. Sulcus shifted to the right cell side, reaching the antapex. Apical hook pointing to the left. APC shifted to the left cell side. Theca smooth with pores. Elongated pusule.

Holotype: the SEM stub containing the type (specimen shown in Figure 4) is deposited at Centre of Excellence for Dinophyte Taxonomy (CEDiT, Wilhelmshaven, Germany), which is part of the Herbarium Senckenbergianum Frankfurt/M. (FR), with the accession number CEDiT2026H215 *A. robustum* sp. nov. Reñé & Hoppenrath.

Collector: Albert Reñé; collection date: 24th July 2017.

Molecular information: nuclear ribosomal SSU (PZ377045‐PZ377046) and LSU (PZ399186‐PZ399187) sequences.

Etymology: From Latin *robustum*; referring to the robust morphology of the species.

Type locality: Castelldefels beach, Catalonia, NW Mediterranean Sea (41°15′35″N, 1°55′51″E).

Habitat: marine, benthic, sandy sediment.

PhycoBank Registration http://phycobank.org/107466.

Plate formula: APC, 4′, 2a, 6″, 5–6?c, 2+?s, 6‴, 2⁗. Length: 24–40 μm. Width: 21–30 μm. Mean L:W ratio = 1.2 (*n* = 10). The cells were heterotrophic, but the feeding behavior has not been observed. The cells were roundish (conical anterior and slightly narrowing, “triangular” posterior) (Figure 3A) and obliquely dorsoventrally compressed (Figure 4C), with an apical hook pointing to the left cell side (Figures 3B and 4A,D). A large nucleus was located in the left side of the hyposome (Figure 3B,D). An elongated pusule was visible at the centre of the cell (Figure 3C). The epitheca was larger than the hypotheca, and slightly wider (Figure 4B). The submedian cingulum was complete and slightly descending, with a first inclining (left ventral), then nearly horizontal to slightly declining (dorsal) and finally slightly declining (right ventral) path—giving it a V‐shape in ventral view (Figures 3E and 4A,B). The sulcus had no extension into the epitheca and reached the antapex. The first apical plate (1′) was narrow and elongated, running from the cingulum to the apex and occupied a central position. The second apical plate (2′) was ventral, large and pentagonal. The third apical plate (3′) was smaller, lateral. The fourth apical plate (4′) was large and elongated, running from the right side of the cell to the apex. It bent to the left, forming an apical hook, that covered the Po. Two anterior intercalary plates were present. The first anterior intercalary plate (1a) was small, and situated laterally, below plate 3′. The second anterior intercalary plate (2a) was large, hexagonal and dorsally located, being in contact with plate 1a. It possessed six precingular plates. The first and second ones (1″ and 2″) were situated ventrally. The third to fifth (3″–5″) were dorsal, and the sixth one (6″), which was the largest one, was located in the ventral right side of the cell. Plate 1″ had a prominent ventral precingular list extension. The number of cingular plates could not be determined with certainty, but at least 6 plates were clearly observed. Plates c1 and c2 were short and ventral. Plates c3–c5 encircled the cell from left lateral, dorsal and right lateral. The plate c6 was ventral, being in touch with the right sulcal plate (Sd = peduncle cover plate PC), which formed a list with pointing (“spine”‐like) posterior end (Figures 3C and 4A,B). A left sulcal list formed by the first postcingular and antapical plates (1‴ and 1⁗) was wider posterior with a rounded flap‐like extension (part of 1⁗) (Figures 3C and 4A,B). The six postcingular plates ran aligned to the precingular ones. The first and second (1‴–2‴) were ventral. The third to fifth (3‴–5‴) were dorsal, and the small sixth one (6‴) was located in the ventral right side of the cell. The first antapical plate (1⁗) was small, located ventrally, and it formed a characteristic small list (flap‐like extension). The second antapical plate (2⁗) was large and encircled the antapex of the cell. Thecal plates were smooth with pores of one size class, which could be dense or more scattered (Figure 4A,B). Pre‐ and postcingular plates had a dense row of pores at their cingular margin (Figure 4B,C).

#### 3.2.2 *Aduncodinium* cf. *robustum* Pachena Beach Canada (Figure 5)

**FIGURE 5:**
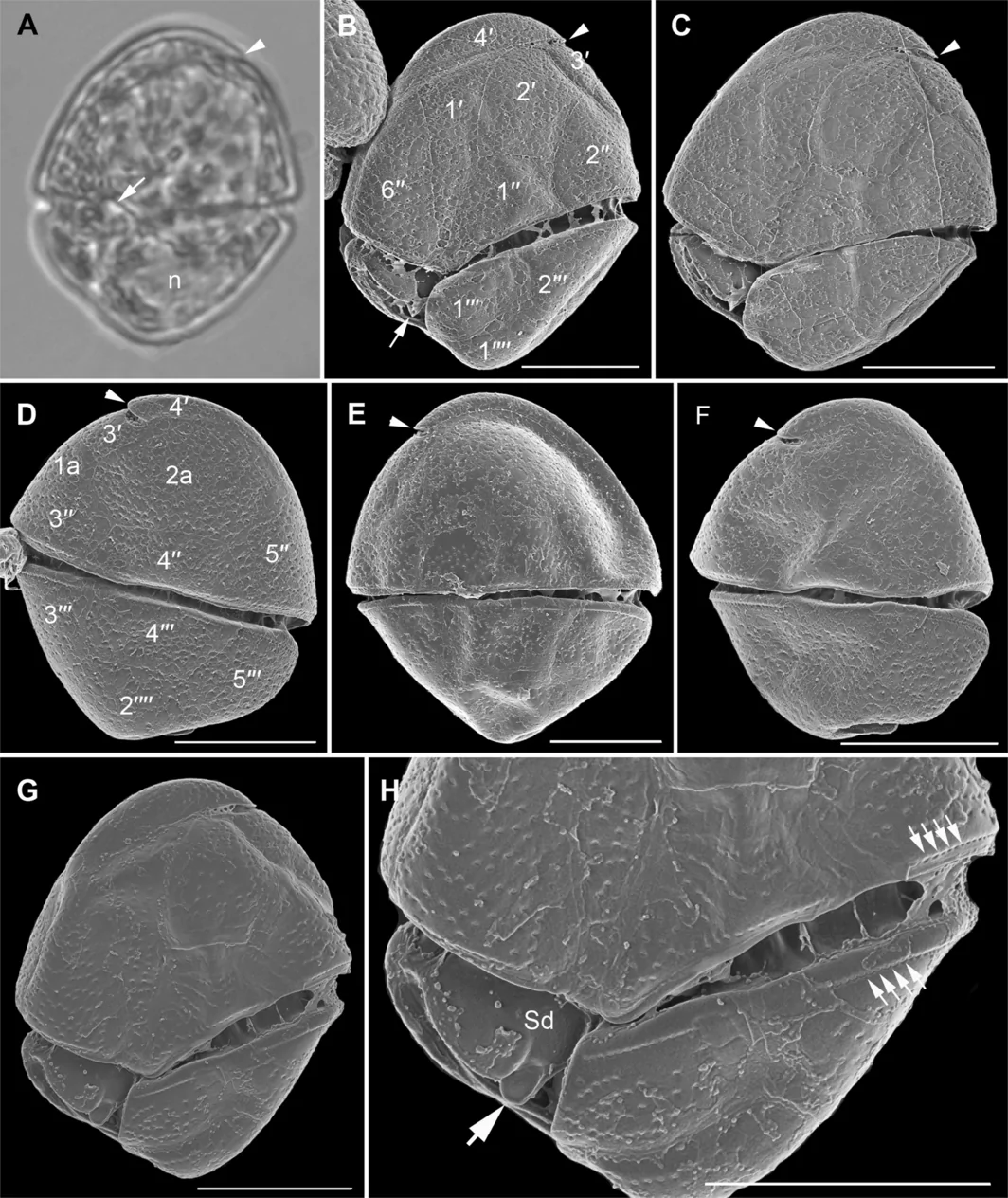
Light microscopy (A) and scanning electron microscopy (B–H) images of *Aduncodinium* cf. *robustum* from Pachena Beach, Canada. (A–C, G, H) Ventral view. (D–F) Dorsal view. Arrowheads indicate the apical hook. Arrows indicate the pointing list in sulcal plate. Correlative small arrows in (H) indicate the rows of pores at the cingular margins. Scale bars = 10 μm.

Cells roundish (conical anterior and narrowing, “triangular” posterior), oblique dorsoventrally compressed, 21–31 μm long, 19–28 μm wide (*n* = 2). Nucleus located in the left side of the hyposome. The epitheca was larger than the hypotheca and slightly wider. Apical hook pointing to the left‐dorsal cell side and cingulum V‐shaped in ventral view. Similar to *A. robustum* but more pronounced narrowing, “triangular” posterior cell end and narrow left sulcal list only formed by the first postcingular plate, not wider posterior and without rounded flap‐like extension (Figure 5E). Plate 1″ without prominent ventral precingular list extension (Figure 5G,H). Right sulcal plate with (acute or blunt) posterior pointing left list that covers the sulcal centre (Figure 5B,H). Likely a round pusule (Figure 5A). Large first antapical plate (1⁗) ventral and the second antapical plate (2⁗) only dorsal. Plate formula: APC, 4′, 2a, 6″, ?c, 1+?s, 5‴, 2⁗. Thecal plates were smooth with scattered pores of one size class (Figure 5G,H). Pre‐ and postcingular plates had a dense row of pores at their cingular margin (Figure 5H).

#### 3.2.3 Aduncodinium glandula

The species has been described in detail from Korea and Japan (Kang et al. 2015; Reñé et al. 2025) and molecular phylogenetic data are available. Cells 11.5–23.5 μm long. Plate formula: APC, 4′, 2a, 6″, 6c, 4s, 5‴, 2⁗. Thecal plates smooth with pores of one size class. Pre‐ and postcingular plates had a dense row of pores at their cingular margin. Without precingular and left sulcal list. Right sulcal plate with short left list. Two connected anterior intercalary plates.

#### 3.2.4 *Aduncodinium* cf. *glandula* Sylt, Germany (Figure 6)

**FIGURE 6:**
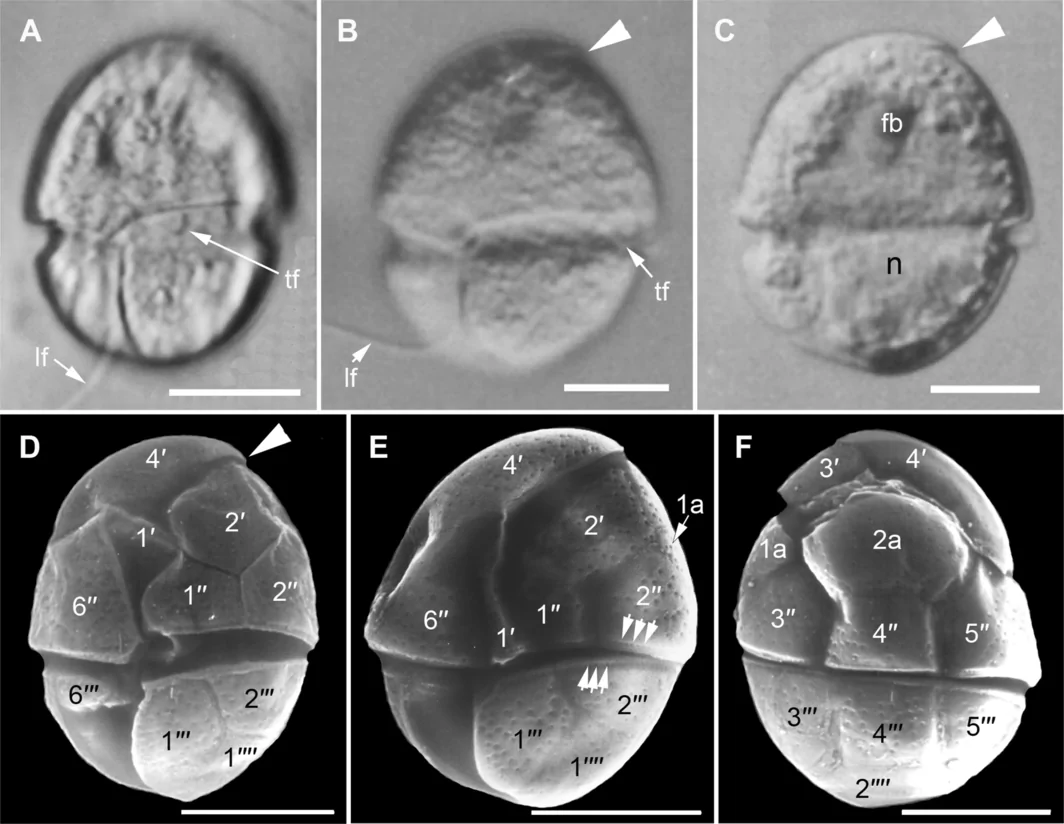
*Aduncodinium* cf. *glandula* Sylt, Germany. (A–C) Light microscope images of cells in ventral view. (D–F) SEM images of cells in ventral (D, E) and dorsal (F) view. Arrowheads indicate the apical hook. Correlative small arrows in (E) indicate the rows of pores at the cingular margins. fb, food body; lf, longitudinal flagellum; n, nucleus; tf, transverse flagellum. Scale bar = 10 μm.

Cells were round (conical anterior and posterior), obliquely dorsoventrally compressed, 20–34 μm long, 15–29 μm wide, making a stocky impression (Figure 6A,D). The episome was larger than the hyposome, dome‐shaped, and slightly wider, with tightly arranged apical hook pointing to the left‐dorsal cell side (Figure 6B,C). The submedian cingulum completely encircled the cell horizontally, with no displacement or descending about half cingular width (Figure 6D,E). The sulcus was offset to the right cell side, had no extension into the epitheca and reached the antapex (Figure 6A,D). Plate 1‴ with left sulcal list. Half of the large nucleus was placed in the left hyposome and projected far into the episome (Figure 6C). The delicate thecal plates were smooth with numerous unevenly distributed pores of one size class and pre‐ and postcingular plates had a dense row of pores at their cingular margin (Figure 6E). The thecal tabulation pattern could only be partly reconstructed: APC, 4′, 2a, 6″, ?c, ?s, 6‴, 2⁗.

#### 3.2.5 *Aduncodinium* cf. *asymmetricum* Ishikari, Japan (Figure 7)

**FIGURE 7:**
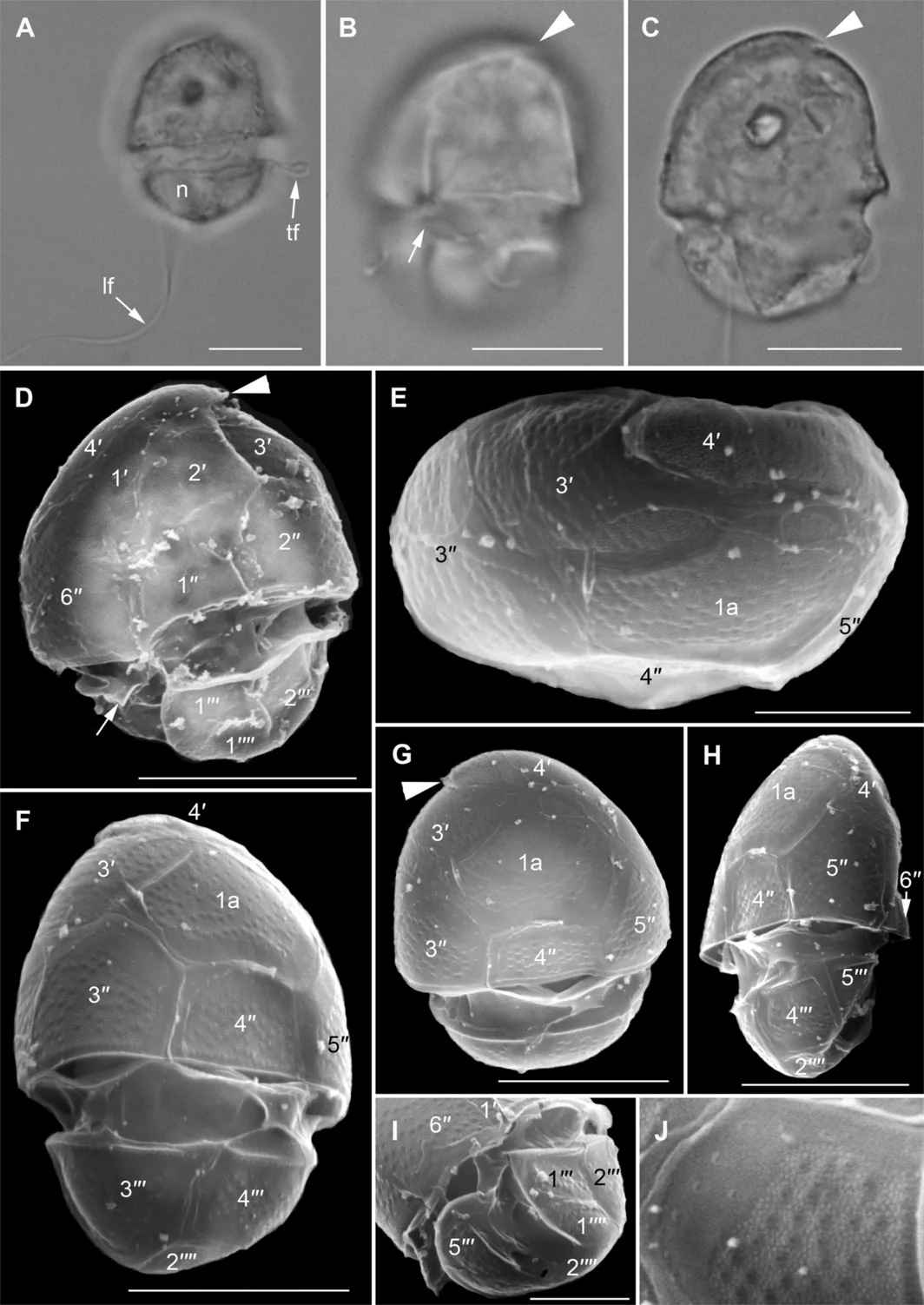
Light (A–C) and scanning electron microscope (D–J) images of *Aduncodinium* cf. *asymmetricum* from Ishikari, Japan. (A) Dorsal view. (B, C) Ventral view. (D) Ventral view. (E) Dorso‐apical view. (F) Left‐dorsal view. (G) Dorsal view. (H) Right lateral view. (I) Antapical view. (J) Detail of the pusticulated ornamentation. Arrowheads indicate the apical hook. Arrow in (B) indicates the transverse flagellum insertion, and in (D) the pointing sulcal list. lf, longitudinal flagellum; n, nucleus; tf, transverse flagellum. Scale bars = 10 μm, except (E, I) = 5 μm.

Cells were 14–20 μm long, 13–16 μm wide (*n* = 3), oval in shape and obliquely laterally flattened (Figure 7A,E). The episome was larger than the hyposome and had an apical hook bent to the left (Figure 7B,D). The small hyposome had a roundish posterior end, with the left side being more prominent than the right one (Figure 7C,F). The nucleus was spherical and located in the posterior part of the cell (Figure 7A). The cingulum was wide, complete and not displaced or slightly descending (about half cingular width) (Figure 7D,I). The sulcus did not extend into the episome (Figure 7D). Plate formula: APC, 4′, 1a, 6″, ?c, ?s, 5‴, 2⁗. The Po plate was covered by an apical hook formed by plate 1′. The 2′ plate was roughly pentagonal and its bottom was convex. The 3′ plate was located on the dorsal side and visible from both, right and left lateral view of the cell. The 1a plate was the largest plate and irregular pentagonal. The 1″, 2″, 4″ and 5″ plates were pentagonal, and plate 3″ was roughly hexagonal. The 1‴ to 4‴ plates were square in shape and in contact with plate 1⁗. Plate 5‴ was triangular and did not contact with plate 1⁗. The 1‴ and 1⁗ (Figure 7D,I). The plate arrangement of the sulcus could not be observed, but a list could be recognized in the sulcal plate (Figure 7D). Thecal plates were thin and easily collapsed and had a fine pusticulate (tightly dotted) ornamentation.

#### 3.2.6 *Aduncodinium* cf. *asymmetricum* Barcelona, Spain (Figure 8)

**FIGURE 8:**
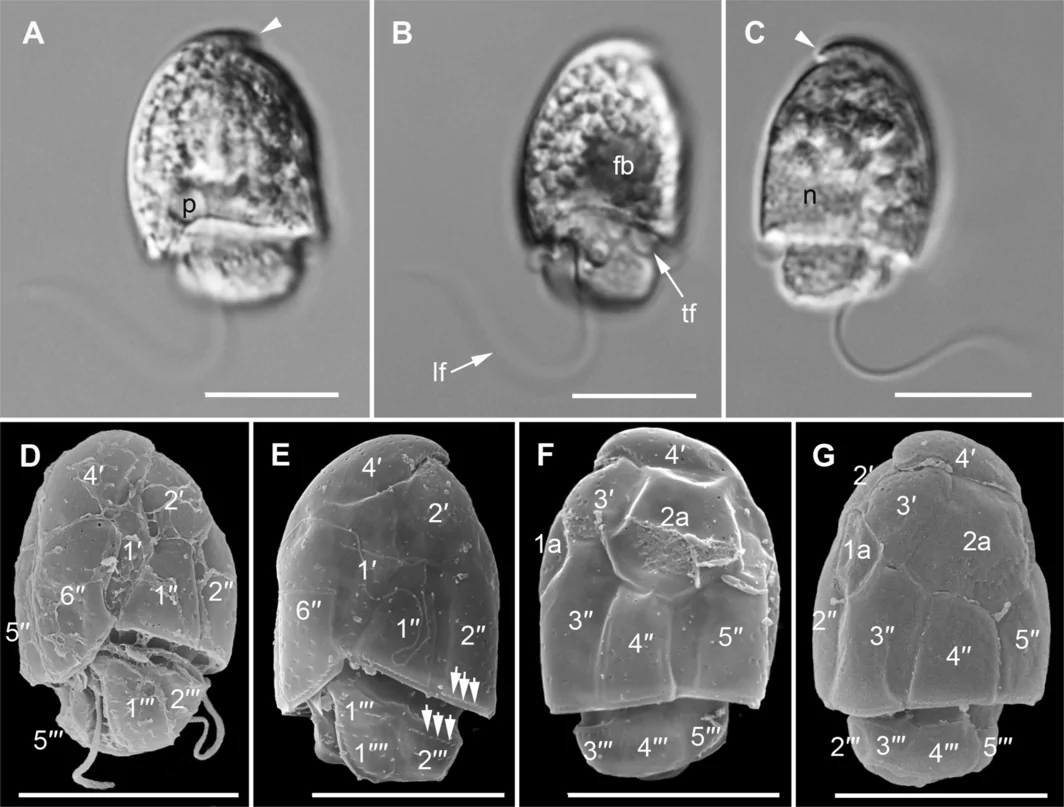
Light (A–C) and scanning electron microscopy (D–G) images of *Aduncodinium* cf. *asymmetricum* from Barcelona, Spain. (A, B, D, E) Ventral view. (C, F, G) Dorsal view. Arrowheads indicate the apical hook. Correlative small arrows in (E) indicate the rows of pores at the cingular margins. fb, food body; lf, longitudinal flagellum; n, nucleus; tf, transverse flagellum. Scale bars = 5 μm.

Cells 20–22 μm long and 12–14 μm wide (*n* = 3), dorsoventrally compressed. Episome elongated (Figure 8A–D). Hyposome small and somehow quadrangular, with flattened antapex (Figure 8B,E). Sulcus shifted to the right lateral side (Figure 8B,D,F). Cingulum descending around one width, forming an inverted V‐shape at the ventral position (Figure 8D,F). Round pusule (Figure 8A). Nucleus elongated, occupying the left side of the cell (Figure 8C). The delicate thecal plates were smooth with scattered pores of one size class and pre‐ and postcingular plates had a dense row of pores at their cingular margin (Figure 8E). The thecal tabulation pattern could only be partly reconstructed: APC, 4′, 2a, 6″, ?c, ?s, 5?‴, 2?⁗. The first apical plate (1′) was narrow and elongated, running from the cingulum to the apex and occupied a central position. The second apical plate (2′) was ventral, and the third apical plate (3′) was smaller and dorsal. The fourth apical plate (4′) was larger, running from the right side of the cell to the apex. It bent to the left, forming an apical hook, that covered the Po. Two anterior intercalary plates were present. The first anterior intercalary plate (1a) was small, and located left lateral, below the 3′. The second anterior intercalary plate (2a) was large, hexagonal and located right dorsal, not in contact with plate 1a. It possessed six precingular plates. The first, second and sixth ones (1″, 2″ and 6″) were situated ventrally. The third to fifth (3″–5″) were dorsal. The number of cingular and sulcal plates could not be determined with certainty. Plate 1‴ relatively small (short) and plate 1⁗ visible on the ventral side. It likely possessed five postcingular plates, and probably two antapical plates, even though 2⁗ could not be observed.

#### 3.2.7 *Aduncodinium* cf. *asymmetricum* Sylt, Germany (Figure 9)

**FIGURE 9:**
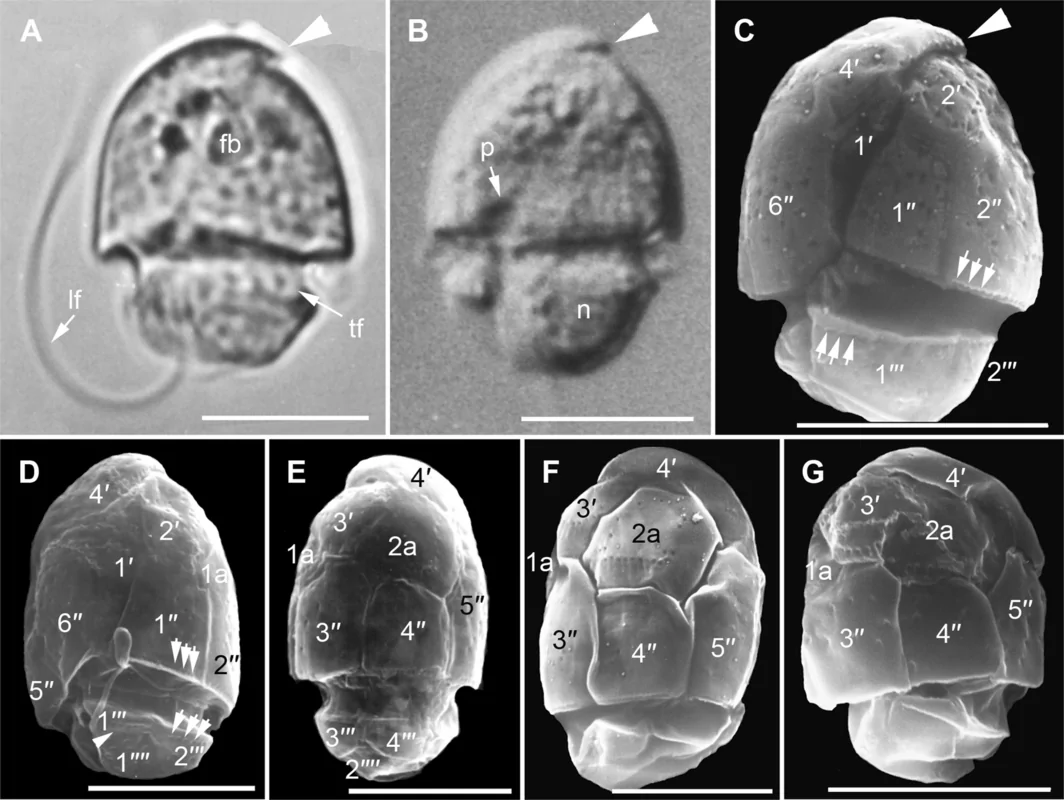
Light microscopy (A, B) and scanning electron microscopy images (C–G) of *Aduncodinium* cf. *asymmetricum* Sylt, Germany, representing the “rounded” morphotype (A–C) and “elongated” morphotype (D–G). Arrowheads indicate the apical hook in (A–C). In (D), it indicates the suture between 1‴ and 1⁗. Correlative small arrows in (C, D) indicate the rows of pores at the cingular margins. fb, food body; lf, longitudinal flagellum; n, nucleus; p, pusule; tf, transverse flagellum. Scale bars = 10 μm.

The species description from Hoppenrath (2000a, 2000b) most likely contained two taxa, one with roundish (Figure 9A–C) and a second with elongated (Figure 9D–G) oblique dorsoventrally compressed cells with narrower, small hyposome, 13–21 μm long and 8–13 μm wide. Dome‐shaped episome around 2 (more rounded morphotype) or 2.5 (narrower, more elongated morphotype) times the length of the hyposome, clearly wider then hyposome, with tightly arranged apical hook pointing to the left‐dorsal cell side. The submedian cingulum completely encircled the cell horizontally, with no displacement (rounded morphotype, Figure 9C) or descending about half cingular width, forming a reverse V‐shape (elongated morphotype, Figure 9D). The sulcus was offset to the right cell side, had no extension into the epitheca and reached the antapex (Figure 9B–D). No or very narrow left sulcal list. The nucleus was placed in the hyposome and projected into the episome. The rounded morphotype had a round pusule (Figure 9B). The delicate thecal plates were smooth with scattered pores of one size class and pre‐ and postcingular plates had a dense row of pores at their cingular margin (Figure 9D). The thecal tabulation patterns could only partly be reconstructed. Rounded morphotype: APC, 4′, 1a?, 6″, ?c, ?s, ?‴, ?⁗, with only one relatively large plate 1‴ and no antapical plate were visible in ventral view. As no dorsal view was documented the number of anterior intercalary plates is only speculative (none visible in ventral view). Elongated morphotype: APC, 4′, 2a, 6″, ?c, ?s, 5‴, 2⁗. The elongated morphotype had two anterior intercalary plates that were not in contact, 1a located left lateral and 2a right dorsal. Plate 1‴ was relatively small (short and horizontally elongated) and plate 1⁗ was visible on the ventral side. This taxon seems to be morphologically identical to the former described species *A*. cf. *asymmetricum* “elongated” from Catalonia, Spain.

#### 3.2.8 *Aduncodinium* cf. *asymmetricum* Wilhelmshaven, Germany (ExtE)

This morphospecies was reported in Reñé et al. (2024). Round to oval cell (conical anterior and posterior), dorsoventrally compressed, 15 μm long and 12 μm wide (*n* = 1). The episome was larger than the hyposome (about 1.5 times its length), dome‐shaped and wider, with tightly arranged apical hook pointing to the left‐dorsal cell side. Hyposome of medium size. Round pusule. No information about the tabulation is available. Similar to the rounded morphotype from Sylt but with relatively longer and more dome‐shaped hyposome.

#### 3.2.9 *Aduncodinium* cf. *asymmetricum* Boundary Bay, Canada (Figure 10A,B)

**FIGURE 10:**
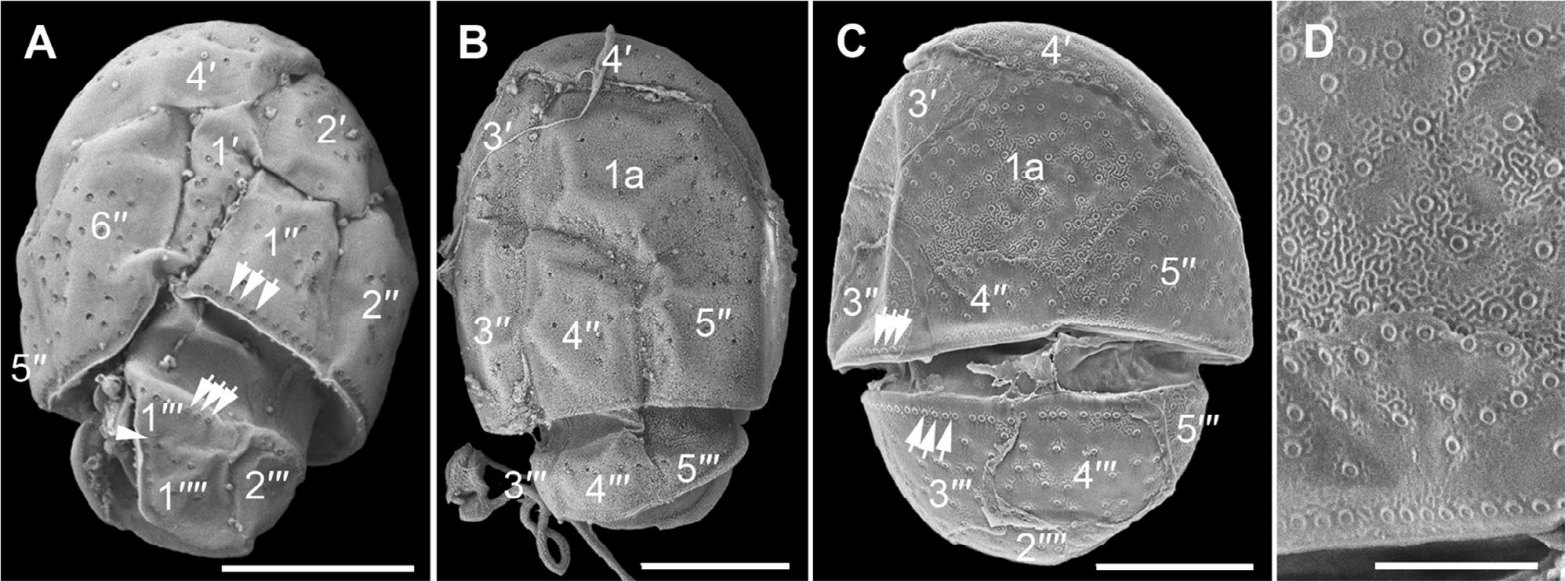
Scanning electron images of *Aduncodinium* cf. *asymmetricum* from Canada. (A, B) Boundary Bay; (C, D) Pachena Beach. (A) Ventral view. (B, C) Dorsal view. (D) Detail of the ridges present in the thecal plates. The arrowhead indicates the suture between 1‴ and 1⁗. Correlative small arrows indicate the rows of pores at the cingular margins. Scale bars = 5 μm, except (D) = 2 μm.

Elongated, dorsoventrally compressed cell, 14–17 μm long and 10–11 μm wide (*n* = 2), with dome‐shaped episome around 2–3 times the length of the hyposome, clearly wider then hyposome (Figure 10A,B). Sulcus maximally shifted to the right lateral side, reaching the antapex and not extending into the episome (Figure 10A). Left (plate 1⁗) and right (plate 4?‴) sulcal list (Figure 10A). Thecal plates were smooth with scattered pores of one size class and pre‐ and postcingular plates had a dense row of pores at their cingular margin (Figure 10A). The thecal tabulation pattern could only partly be reconstructed: APC, 4′, 1a, 6″, ?c, ?s, 4 (5?)‴, 2?⁗.

#### 3.2.10 *Aduncodinium* cf. *asymmetricum* Pachena Beach, Canada (Figure 10C,D)

Round to oval cell (conical anterior and posterior), dorsoventrally compressed, about 17 μm long and 13.5 μm wide (*n* = 1). The episome was larger than the hyposome (about 2 times its length), dome‐shaped and wider, with tightly arranged apical hook pointing to the left‐dorsal cell side (Figure 10C). Hyposome of medium size. Submedian cingulum (Figure 10C). Thecal plates with characteristic ornamentation and pores of one size class (Figure 10D). Pre‐ and postcingular plates had a dense row of pores at their cingular margin (Figure 10C). Uncertain tabulation, likely with APC, 4′, 2?a, and 6″ plates.

#### 3.2.11 *Aduncodinium* spec 1 Barcelona, Spain (Figure 11)

**FIGURE 11:**
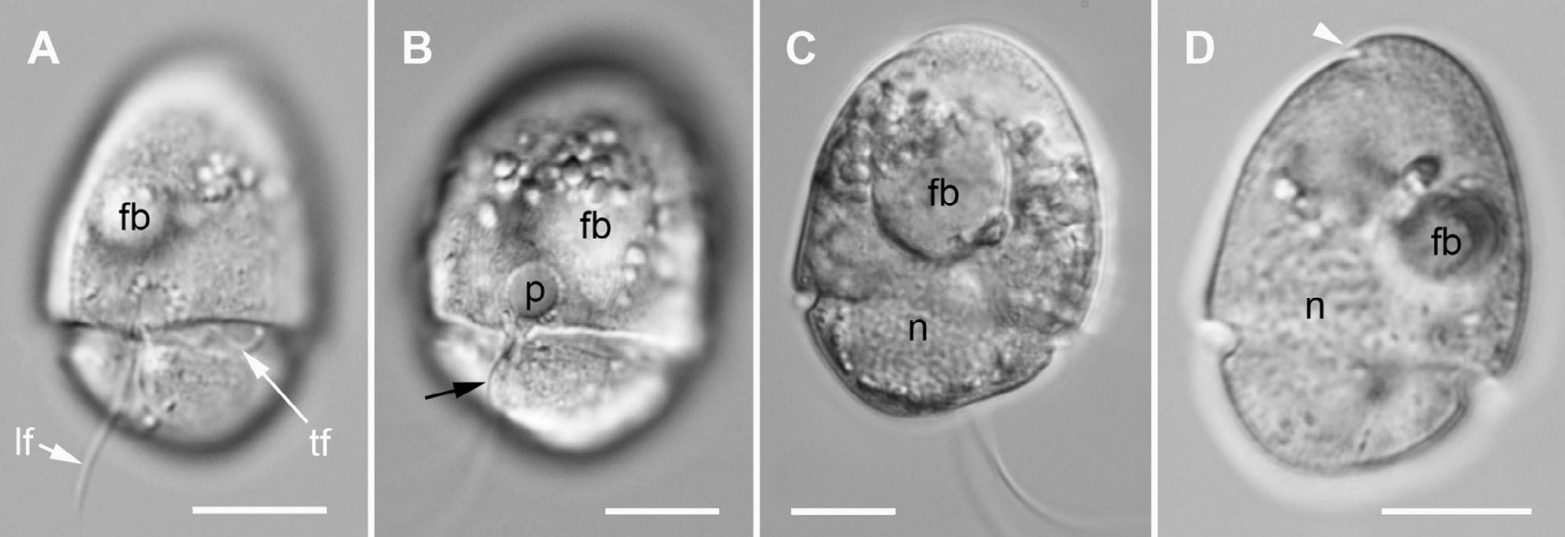
Light microscopy images of *Aduncodinium* spec 1 Barcelona, Spain. (A, B) Ventral view. (C, D) Dorsal view. fb, food body; n, nucleus; p, pusule. The arrow indicates the wing in the postcingular 1′ plate and the arrowhead indicates the apical hook. fb, food body; lf, longitudinal flagellum; n, nucleus; p, pusule; tf, transverse flagellum. Scale bars = 10 μm.

Elongated cell, 30–37 μm long; 21–27 μm wide (*n* = 3), with episome around 2 times the length of the hyposome. Hyposome slightly narrower than the episome, with the left side being more prominent than the right one. Apical plate bent to the left, forming an apical hook (Figure 11C,D). Cingulum almost straight in ventral view (Figure 11A,B). Round pusule (Figure 11A,B). Prominent list in 1⁗ (Figure 11B). Nucleus running vertically at the left side of the cell (Figure 11D). A large yellow body present in the episome (Figure 11A–D).

#### 3.2.12 *Aduncodinium* spec 2 Sylt, Germany (Figure 12)

**FIGURE 12:**
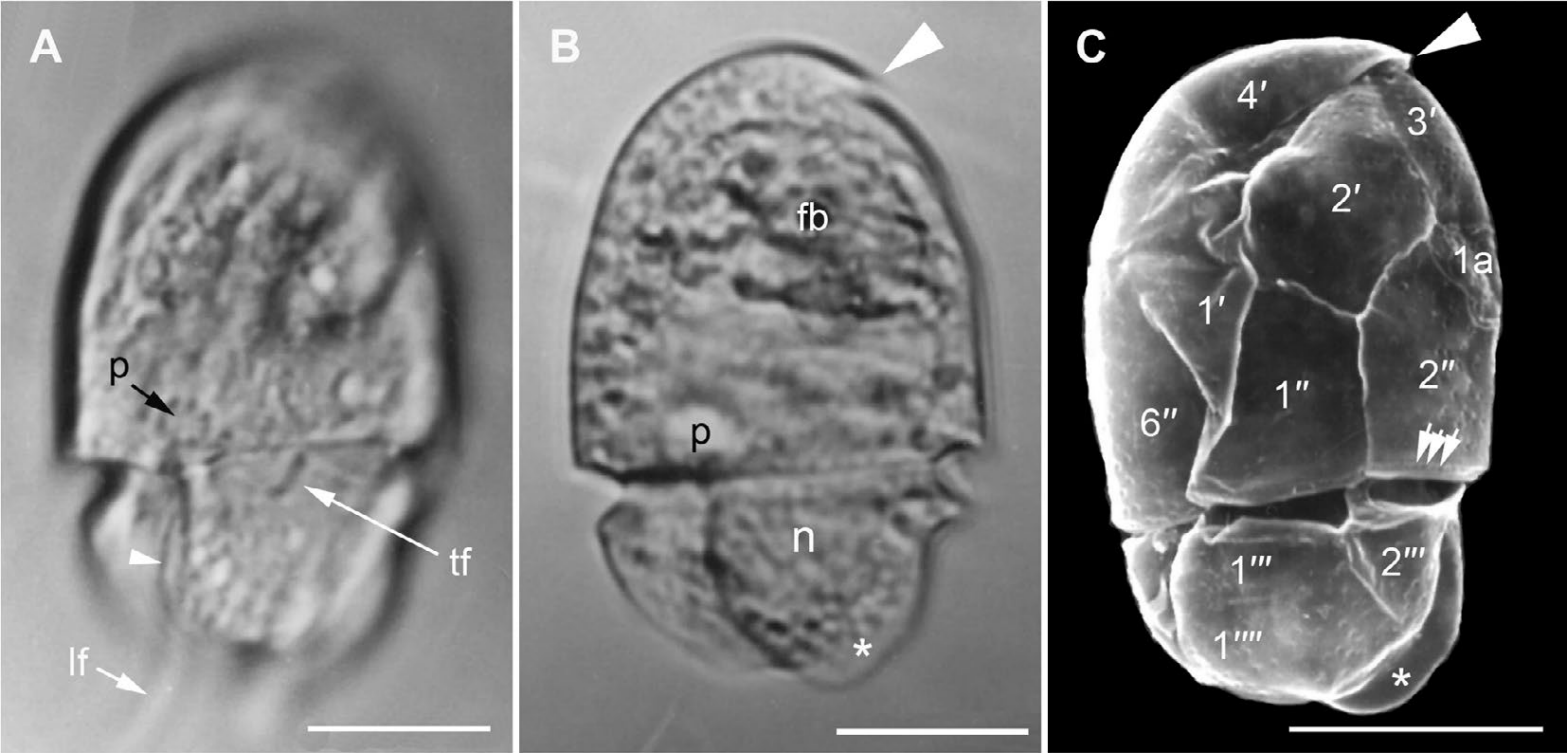
Light (A, B) and scanning electron microscopy (C) images of *Aduncodinium* spec 2 from Sylt, Germany in ventral view. The arrowheads indicate the apical hook. Correlative small arrows indicate the rows of pores at the cingular margins. fb, food body; lf, longitudinal flagellum; n, nucleus; p, pusule; tf, transverse flagellum; *, keel‐like structure. Scale bars = 10 μm.

Elongated, obliquely dorsoventrally compressed cell, 23–34 μm long and 16–20 μm wide, with dome‐shaped episome around 2 times the length of the hyposome, slightly wider (Figure 12B). Tightly arranged apical hook pointing to the left‐dorsal cell side. The cingulum completely encircled the cell horizontally, with no displacement (Figure 12A,B). The sulcus was offset to the right cell side, had no extension into the epitheca and reached the antapex (Figure 12A). Possibly a left sulcal list was formed by plate 1⁗ (Figure 12A). Half of the large nucleus was placed in the left hyposome and projected far into the episome. Round pusule (Figure 12B). The delicate thecal plates were smooth with numerous unevenly distributed pores of one size class and pre‐ and postcingular plates had a dense row of pores at their cingular margin (Figure 12C). The thecal tabulation pattern could only be partly reconstructed: APC, 4′, 2a?, 6?″, ?c, ?s, 5?‴, ?⁗. At the left‐dorsal hyposome a keel‐like structure was observed and it needs to be checked whether it is a constant taxon‐specific feature and which plates form it. It shows high similarity with the former described elongated species 1 from the Mediterranean.

#### 3.2.13 *Aduncodinium* spec 3 Boundary Bay, Canada (Figure 13)

**FIGURE 13:**
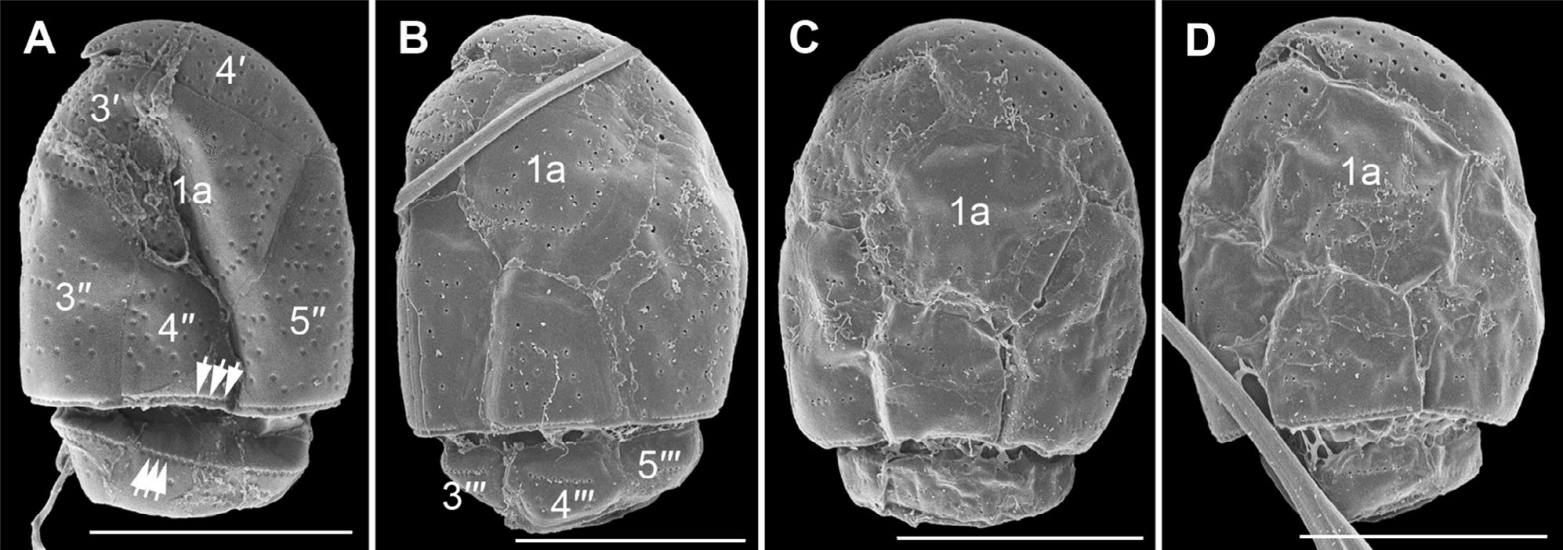
Scanning electron images showing cells of *Aduncodinium* spec 3 from Boundary Bay, Canada in dorsal view. Correlative small arrows indicate the rows of pores at the cingular margins. Scale bars = 10 μm.

Elongated, dorsoventrally compressed cell, 19–23 μm long and 13–15 μm wide (*n* = 4), with dome‐shaped episome around 3 times the length of the narrow hyposome, slightly wider. Thecal plates were smooth with numerous unevenly distributed pores of two size classes (larger pores on plate 4′ in Figure 13D) and pre‐ and postcingular plates had a dense row of pores at their cingular margin (Figure 13A). The thecal tabulation pattern could only be partly reconstructed: APC, 4′, 1a, 6?″, ?c, ?s, 5?‴, ?⁗.

#### 3.2.14 *Aduncodinium* spec 4 Pachena Beach, Canada (Figure 14)

**FIGURE 14:**
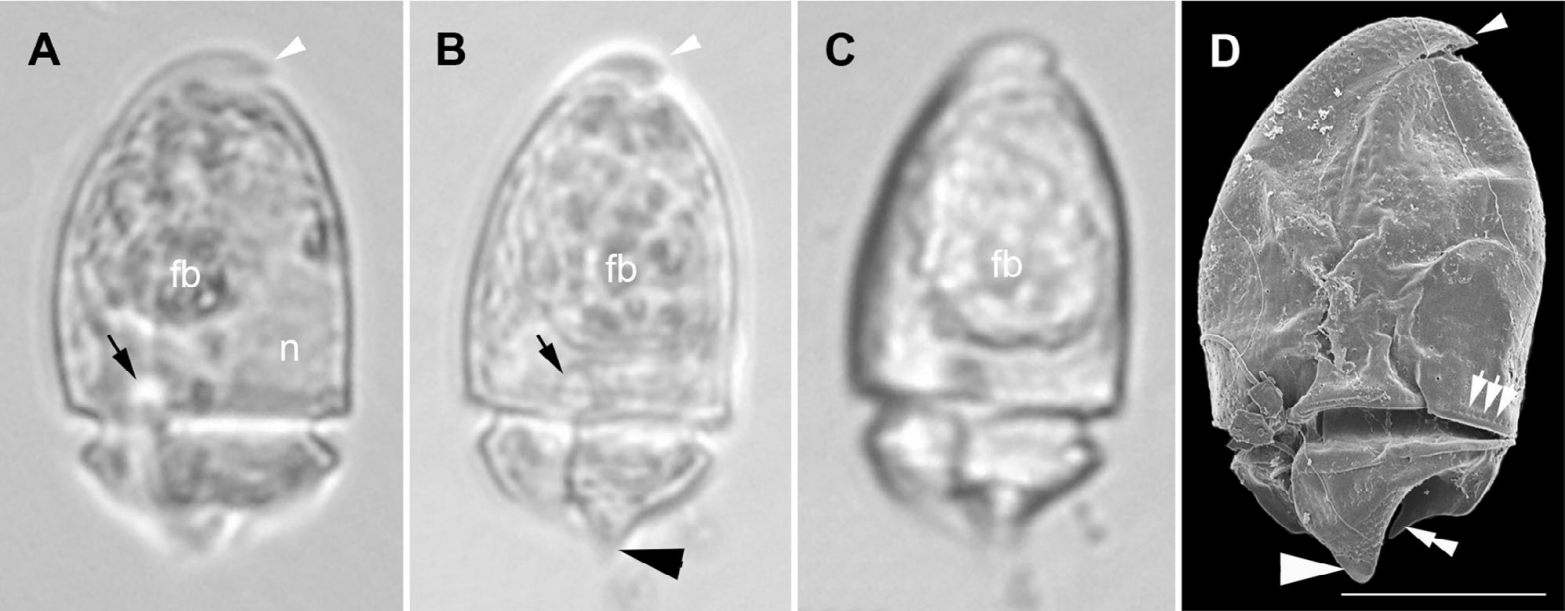
*Aduncodinium* spec 4 from Pachena Beach, Canada in ventral view. (A–C) Light microscope images. (D) Scanning electron image. The arrows indicate the round pusule. The anterior small arrowheads indicate the apical hook. The large arrowheads indicate the posterior spine‐like horn, and the double arrowhead the antapical spine. Correlative small arrows indicate the rows of pores at the cingular margins. fb, food body; *n*, nucleus. Scale bars = 10 μm.

Elongated, obliquely dorsoventrally compressed cell, about 27–40 μm long and 17–20 μm wide (*n* = 3), with dome‐shaped apically narrowing episome around 3 times the length of the hyposome, slightly wider. Apical hook pointing to the left‐dorsal cell side. The cingulum completely encircled the cell horizontally, with no displacement. The sulcus was offset to the right cell side, had no extension into the epitheca and reached the antapex. Nucleus was placed in the left dorsal episome (Figure 14A). Round pusule (Figure 14B). Thecal plates were smooth with numerous unevenly distributed pores of one size class, and pre‐ and postcingular plates had a dense row of pores at their cingular margin (Figure 14D). The thecal tabulation pattern could not be reconstructed. The first antapical plate had ventrally a characteristic shape of a posterior pointing spine‐like horn (Figure 14B,D). An antapical spine—not recognizable in the light microscope—was likely part of a second antapical plate or a dorsal postcingular plate (Figure 14D).

#### 3.2.15 *Aduncodinium* spec 5 (Figure 15)

**FIGURE 15:**
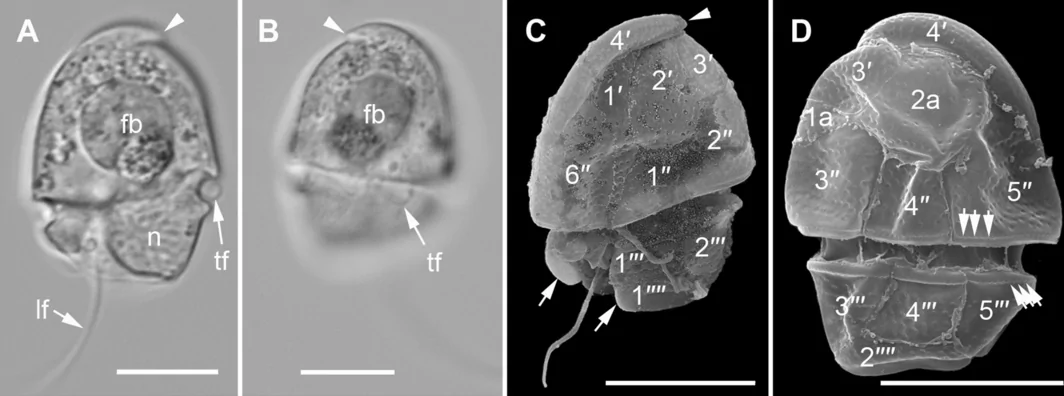
Light microscopy (A, B) and scanning electron microscopy (C, D) images of *Aduncodinium* spec 5 from L'Estartit, Spain. (A, C) Ventral view. (B, D) Dorsal view. The arrowheads indicate the apical hook. The arrows indicate the rounded flap‐like extension in 1″, and the wing in the sixth postcingular plate. Correlative small arrows indicate the rows of pores at the cingular margins. fb, food body; lf, longitudinal flagellum; n, nucleus; tf, transverse flagellum. Scale bars (A, B) = 5 μm, (C, D) 10 μm.

Cells 19–24 μm long; 15–17 μm wide (*n* = 3). Hyposome almost the same width than the episome (Figure 15A). Episome ovoid (dome‐shaped), with almost twice the length of the hyposome. Sulcus shifted to the right. Hyposome with the left side more prominent and quadrangular. Right side smaller and triangular (Figure 15A,B,D). Nucleus in the left hyposome. Ingestion body present. Round pusule (Figure 15A). First apical plate central and narrow. Second apical plate on the ventral left and third apical plate in the left lateral side of the cell. Fourth apical plate bent to the left forming a hook (Figure 15C). Two anterior intercalary plates in contact, 1a placed between 3′ and 3″, 2a large, dorsally located (Figure 15D). First precingular plate horizontally elongated. Second and sixth precingular plates large and ventral. Third to fourth precingular plates dorsal. Cingulum almost straight, slightly descending (Figure 15A,C). Five (probably six) postcingular plates and two antapical plates (Figure 15C,D). The number of cingular and sulcal plates could not be determined. Two lists in the sulcal area, one rounded and one with pointing posterior end (Figure 15C). Rounded flap‐like extension in 1⁗. Wing in the sixth postcingular plate and sulcal plates (Figure 15C). Antapical keel part of the second antapical plate (Figure 15D). Dense row of pores at their cingular margin in the pre‐ and postcingular plates (Figure 15D). Plate formula: APC, 4′, 2a, 6″, ?c, ?s, 5–6‴, 2⁗.

As shown in the previous section, the SSU and/or LSU rDNA sequence could be obtained for some of the morphospecies observed. Two additional sequences are provided, one corresponding to *Aduncodinium* cf. *asymmetricum* from Canada (SSU rDNA) and one to *Aduncodinium* sp. from Catalonia (LSU rDNA). Unfortunately, no images could be obtained. Consequently, no morphological details of sequenced species can be provided and they cannot be assigned to any of the morphospecies described here.

## 4 Discussion

### 4.1 Overview of Morphospecies Observed

*Katodinium* species showing *Aduncodinium*‐like characters are recurrently reported in the literature, showing they are common and widely distributed. In most cases the identification is solely based on the external morphology of the cells, and most specimens are reported basically under the species names *K. glandula* and *K. asymmetricum* (e.g., Dodge 1982; Gómez and Artigas 2014; Larsen and Patterson 1990). However, the images available in the literature show differing morphologies, suggesting they likely represent different species. In any case, the information provided is scarce and not proving helpful for their discrimination. In this study, morpho‐molecular information of several *Aduncodinium*‐like morphospecies is provided, showing similar plate arrangements and close phylogenetic relationships. The results obtained suggest the existence of at least eight different *Aduncodinium*‐like species based on the molecular information obtained. This number can be increased to 16 species when including the different morphospecies observed. These species have been organized in different morphogroups based on their characteristics.

The *A. glandula* morphogroup comprises (large) species, with the hyposome almost the same width as the episome. They have a smooth theca, 2a connected, 5‴ or 6‴, straight or V‐shaped cingulum. In contrast, the *Aduncodinium asymmetricum* morphogroup taxa comprise smaller species showing a narrower hyposome compared to the episome. They have smooth or ornamented theca, 1a or 2a without contact, 5‴ (or 4‴)?, straight or reverse V‐shaped cingulum. Finally, the *A*. “*elongatum*” morphogroup is represented by medium‐sized to large species with relatively small and narrower hyposome and elongated episome (longer compared to the width as in the *A. glandula* group). They have smooth theca, 1a (or 2a)?, all straight cingulum? In any case, these morphogroups have not yet been recovered in the molecular phylogenies, likely because of the small taxon sample. Additionally, the SSU rDNA sequences do not seem to provide robust information about the phylogenetic relationship between *Aduncodinium* representatives.

#### 4.1.1 *Aduncodinium glandula* Morphogroup

*Aduncodinium glandula* was originally found in sandy sediments from Port Erin, Isle of Man (Herdman 1924). The cells were described to be ovoid and flattened, with episome helmet‐like and apex produced into a sharp point; with post‐median cingulum without displacement; with hyposome half the height of the episome and not quite as wide; with sulcus running obliquely to the left hyposome side; with spherical nucleus in the middle of the cell, but drawn also in the left hyposome area reaching into the episome; and cell length of 20–35 μm (Herdman 1924). The cells were colorless and red or yellow bodies were often present. The identity of *Aduncodinium glandula* has recently been determined (Kang et al. 2015; Reñé et al. 2025), because other than the fact that it initially was not recognized as thecate, all characteristic features of the specimens studied from Korea and Japan were in agreement with the original description. However, this interpretation needs verification by re‐investigating the species at its type locality as the sampling sites were far distant. The morphospecies has been repeatedly reported in the literature, it has been detected in the British Isles (Dodge 1982) and Northern Scotland (Dodge 1989), the North German Wadden Sea (Hoppenrath 2000b), in Kuwait, Arabian Gulf (Al‐Yamani and Saburova 2010), and in Wimereux, English Channel, close to the type locality (Gómez and Artigas 2014). Dodge (1982, 1989) gave only a rather schematic drawing and listed the species, so that the identifications cannot be judged. The taxon recorded from Germany (Hoppenrath 2000b) has been re‐evaluated in this study and as it shows slight differences to *A. glandula* sensu Reñé et al. (2025)—larger size, likely six postcingular plates and a left sulcal list—it is treated as possible separate species, *A*. cf. *glandula*. Specimens depicted from Kuwait (Al‐Yamani and Saburova 2010) show similarities to the German *A*. cf. *glandula*, the cell size fits in the size range of German specimens, but the presence of two anterior intercalary plates is not clear (Figure S1e, their figure 42 h). Cells observed in a sample from Barcelona, Catalonia, looked similar in the light microscope to the taxon from Kuwait (Figure S1). Their cell size was similar, although slightly smaller for Mediterranean cells (23–25.5 μm long, 17–19.6 μm wide and 18.5–19.5 μm long, 15–16 μm wide, respectively). Specimens documented as “*K*.” *glandula* by Gómez and Artigas (2014) in their Figure 8S–V probably correspond to two different species, since the specimen of Figure 8V is clearly smaller than the others. Besides *A. robustum* , which will be discussed below, a further species can be classified within the *A. glandula* morphogroup; *Aduncodinium* cf. *robustum* was discovered in Pachena Beach samples, Canada, and differed from the new Mediterranean species by slightly smaller cell sizes, only five postcingular plates and lacking the precingular list extension of plate 1″ and also having none or only a very reduced left sulcal list without extension.

#### 4.1.2 *Aduncodinium* “*asymmetricum*” Morphogroup

The presence of “*Katodinium*” *asymmetricum* has also been reported recurrently in the literature. The molecular information available for *A*. cf. *asymmetricum* specimens (Reñé et al. 2024, 2025), and new data generated in this study suggest the existence of cryptic species. As such, they conform a species‐complex. Unless a detailed characterization of their morphological features is available, they cannot be distinguished with certainty, even though slight differences could be determined for some of the morphospecies observed in this study. Furthermore, it raises the question about the real identity of the type of the species, described from Nieuwpoort in Belgium. *Gymnodinium asymetricum* was named as a new species by Massart (1920), without a written morphological characterization but with drawings showing the general cell shape with large episome, much smaller and narrower hyposome, the sulcus shifted to the right cell side separating the hyposome in a small right and larger (also longer) left side, with vacuoles and granules in the episome, and the nucleus located in the left hyposome extending into the episome (Massart 1920; figure 22). It will be impossible or at least difficult to decide which of the morphotypes represents the true *K. asymmetricum* until it is re‐discovered and investigated at its type locality. Biecheler (1952) provided a more detailed description of “*Massartia asymetrica*,” but failed to recognize it as a thecate species. Such uncertainty complicates the transfer of *K. asymmetricum* to the genus *Aduncodinium*, even though it can be affirmed with certainty that it will belong to the sensu lato genus as presented herein and not to the athecate genus *Katodinium*.

The species complex has been documented in Belgium (Biecheler 1952; Conrad and Kufferath 1954; Massart 1920), Denmark (Larsen 1985), UK (Carter 1937; Dodge 1982), the North German Wadden Sea (this study; Hoppenrath 2000b; Reñé et al. 2024), France (Gómez and Artigas 2014), or the Mediterranean Sea (this study). It has also been documented in Australia (Al‐Qassab et al. 2002; Larsen and Patterson 1990), or in the USA (Campbell 1973; Hulburt 1957).

As previously stated, most reports of *Aduncodinium*‐like taxa from the literature were assigned to *K. glandula* or *K. asymmetricum* . However, some of them can be re‐considered. The report of *K. glandula* from Australia published in Larsen and Patterson (1990) probably represents *K. asymmetricum* . Gómez and Artigas (2014) reported *K. asymmetricum* in sediments from the English Channel, France. However, different morphospecies can be recognized. The cell from their Figure 8W, reported as “*K*.” *glandula* is clearly different from *A. glandula* specimens and likely represents the same morphospecies as *Aduncodinium* cf. *asymmetricum* Barcelona, Spain with elongated episome from this study. Likewise, the cells from their Figure 8X,Y are reported as *K. asymmetricum* but the *Aduncodinium*‐like specimens show a quadrangular hyposome, like *Aduncodinium* spec 5 from this study, observed in the Mediterranean Sea.

#### 4.1.3 *Aduncodinium* “*elongatum*” Morphogroup

*Aduncodinium* spec 1 from the Mediterranean Spanish coast shows a close affinity to *A*. spec 2 specimens from the Wadden Sea in Germany, originally reported as *Katodinium* spec 1 (Hoppenrath 2000b). It also probably corresponds to the same species as the specimen from the Danish Wadden Sea (Larsen 1985), even though it was identified as *K. glandula*. That morphospecies shows resemblance to *Aduncodinium* spec 4 from Canada, but the cells of this latter are more elongated and show a pointed posterior end on the left side of the cell. To the best of our knowledge, such morphospecies has been reported in the published literature once. Only Baillie (1971) in his MSc thesis described and depicted it as *Katodinium* sp. 1 (his plate 1, Figure 2a and plate 9, Figure 1a), unfortunately mixed with observations/images of a second taxon, likely a *Heterocapsa* species. It was observed in samples from Brady's and Pachena Beach and cells were 26–30 μm long.

#### 4.1.4 Similarities of *Aduncodinium robustum* With Closest Species

As previously mentioned, we integrate *A. robustum* within the *A. glandula* morphogroup. As such, it is a species with the hyposome almost the same width as the episome. Two similar morphospecies were observed, the second one identified as *A*. cf. *robustum*. They were mainly distinguished by the shape of the hyposome posterior end and the absence of list extensions. The presence of a pusule was consistently observed in most *Aduncodinium*‐like species, showing a round shape. However, it was elongated in *A. robustum* , representing a diagnostic feature. Finally, *A. robustum* possesses six postcingular plates, while only five could be determined for *A*. cf. *robustum*. *A. robustum* shows significant differences with *A. glandula*. Cells of *A. robustum* are roundish while *A. glandula* shows a more elongated episome. Again, *A. robustum* possesses six postcingular plates, while *A. glandula* has only five. The cingulum is straight and only slightly descending in *A. glandula*. In contrast, it is V‐shaped in *A. robustum* . Another significant difference is the presence of a precingular and a prominent list in 1⁗, which are absent in *A. glandula*. To the best of our knowledge, this morphospecies has never been reported before in the literature.

#### 4.1.5 Occurrence of *Aduncodinium*‐like Representatives

The majority of morphospecies detected in this study correspond to sand‐dwelling coastal dinoflagellates. Some reports from the literature correspond to the observation of planktonic water samples, for example, Kang et al. (2015), even though they were obtained in coastal, shallow environments. It suggests that *Aduncodinium* representatives are tychoplanktonic organisms, that is, benthic taxa that appear in the water column through disturbances of the benthic habitat, or in the case of these predatory taxa, following the presence of potential prey in the planktonic compartment. Consequently, it indicates that the diversity inhabiting the benthic compartment, and in particular marine sandy sediments, needs to be further explored if we want to determine the real diversity of many dinoflagellate genera, including the genus *Aduncodinium*.

#### 4.1.6 *Aduncodinium*‐like Taxa Compared to Pfiesteriaceans

The phylogenetic results obtained from SSU and LSU rDNA trees suggest *Aduncodinium* is a sister lineage to all other Pfiesteriaceae. The (heterotrophic) pfiesteriacean genera share the same hypothecal tabulation (5‴ 0p 2⁗) but differ in epithecal details (Table 3). Most genera have four apical plates, but *Cryptoperidiniopsis* has five. *Cryptoperidiniopsis* has no anterior intercalary plate and six precingular plates, like *Tyrannodinium*. The genera with one anterior intercalary plate differ in the number of precingular plates, *Pfiesteria* having 5″, *Pseudopfiesteria* having 6″, and *Amyloodinium* having 7″. *Stoeckeria* and *Luciella* both have two anterior intercalary plates but differ in their arrangement—connected in *Stoeckeria* and separated in *Luciella*—and the number of precingular plates (seven in *Stoeckeria* and six in *Luciella*).

**TABLE 3:** Comparison of morphological and ecological data between Pfiesteriaceae genera.

Taxon	Tabulation	Cell sizes		Habitat
		Length [μm]	Width [μm]	
*Pfiesteria piscicida*	APC 4′ 1a 5″ 6c 6+s 5‴ 0p 2⁗	5–18 (60?)		Estuarine waters, plankton
				Brackish/marine plankton
***(Pseudo)Pfiesteria***				
*P. shumwayae*	APC 4′ 1a 6″ 6c 6+s 5‴ 0p 2⁗	10–25		Brackish/marine plankton
				Mesohaline estuaries
*Stoeckeria algicida*	APC 4′ 2a 7″ 6c 6s 5‴ 0p 2⁗	7.3–20.8	2.7–17.4	Brackish/marine plankton
***Cryptoperidiniopsis***				
*C. brodyi*	APC 5′ 0a 6″ 6c 6+s 5‴ 0p 2⁗	6.3–10.6	5.4–9.5	Estuarine waters, plankton
				Also sediment samples
***Luciella***				
*L. masanensis*	APC 4′ 2a 6″ 6c 6+s 5‴ 0p 2⁗	9.6–15.7		Estuarine waters, plankton
*L. atlantis*	APC 4′ 2a 6″ 6c 6+s 5‴ 0p 2⁗	9.5–21.4		Estuarine waters, plankton
*Tyrannodinium*	APC 4′ 0a 6″ 6c 6+s 5‴ 0p 2⁗	20–37		Freshwater plankton
*Amyloodinium*	APC 4′ 1a 7″ 6–8c ?s 5‴ 0p 2⁗	6.1 ± 0.8	11.7 ± 0.5	Parasite of marine fish
***Paulsenella***				Ectoparasites of diatoms
				Marine plankton
*P. chaetoceratis*	Unknown	12.0–15.0	10.0–13.0	On *Chaetoceros decipiens*
*P. kornmannii*	Unknown	15.0–19.0	12.0–15.0	On *Eucampia zodiacus*
*P. vonstoschii*	Unknown	14.0–18.0	12.0–14.0	On *Helicotheca tamesis*
				On *Mediopyxis helysia*
***Aduncodinium***				
*A. glandula*	APC 4′ 2a 6″ 6c 4s 5‴ 0p 2⁗	10.2–29.2	7.7–24.9	Brackish/marine waters
				Sediment/plankton

Within the above‐described diversity of potential *Aduncodinium* species quite some tabulation variability has been observed. All taxa had four apical plates (with an apical hook as part of plate 4′ pointing to the left‐dorsal cell side covering the APC) and six precingular plates (6″). They varied in the number and arrangement of the anterior intercalary plates (1a or 2a, 2a connected or separated), like the other pfiesteriaceans. In contrast, the number of precingular plates seems to be constant from what is known so far, but the postcingular plate number varied, 5‴ or 6‴ and maybe also only 4‴ (Tables 1 and 2) and the arrangement of antapical plates differed. A characteristic feature of Pfiesteriaceae is the presence of a peduncle cover plate (PC), covering the proximal part of the sulcus. It has been found in all species examined in detail (Calado et al. 2009). This plate is also present in *Aduncodinium*, but referred to as Sd. Swarming swimming behavior has also been suggested as characteristic of all Pfiesteriaceae (Calado et al. 2009). It has been observed in *Speroidinium fungiforme* (Spero and Morée 1981) and *A. glandula* (Jang et al. 2016; Reñé et al. 2025), but not in other *Aduncodinium*‐like taxa (Seaborn et al. 2006; this study). Thus, detailed observations of the swimming behavior of *Aduncodinium* specimens must be conducted to determine this feature. Finally, cell division and life‐cycle stages have not been examined in this study, impeding the determination of the formation of cysts, like other pfiesteriaceans such as *P. piscicida* , *Pseudopfiesteria shumwayae*, or *Cryptoperidiniopsis brodyi* (Calado et al. 2009; Litaker et al. 2002; Parrow and Burkholder 2003a, 2003b).

In the light of the characters used to separate genera in other Pfiesteriaceae taxa, it can be discussed whether the tabulation differences in the observed *Aduncodinium* morphospecies are species features or represent generic level traits. Until further, more complete datasets are available, we recommend using a wide genus concept (*Aduncodinium* s.l.) and to keep the possibility of a genus complex in mind, like for *Amphidiniopsis* (Reñé et al. 2020).

## Funding

This work was supported by Ministerio de Ciencia e Innovación, PID2023‐150672NB‐I00. Deutsche Forschungsgemeinschaft, 3267/1‐1. National Science Foundation, EF‐0629624.

## Supporting information


**Figure S1:** Light microscopy images of *Aduncodinium* cf. *robustum* from (A, B) Castelldefels, Spain, and (C–F) Kuwait (Al‐Yamani and Saburova 2010). Arrowheads indicate the apical hook. The arrow indicates the left sulcal list. n, nucleus; p, pusule. Scale bars = 10 μm.

**Table S1:** Species affiliated to the genus *Katodinium* and its current classification (according to AlgaeBase, June 2025).

**Table S2:** List of primers used for PCR amplification of SSU and LSU rDNA sequences.

## Data Availability

The data that support the findings of this study are available from the corresponding author upon reasonable request.
