# Supplementary material for: Exploration of Overlooked Aduncodinium‐Like (Pfiesteriaceae, Dinophyceae) Diversity
Source: J Eukaryot Microbiol. 2026 Aug 21;73(5):e70116. doi: 10.1111/jeu.70116 (PMC13498770; doi:10.1111/jeu.70116)
Supplement: Supplementary file 1 [file JEU-73-e70116-s001.docx]

Table S2. List of primers used for amplification of SSU and LSU rDNA sequences.

| Primer | rDNA gene | Direction | PCR round | Sequence (5'-3') | Reference |
| --- | --- | --- | --- | --- | --- |
| EK-82F | SSU | Forward | 1st & 2nd | GAA ACT GCG AAT GGC TC | López-García et al. (2001) |
| 28S-1611R | LSU | Reverse | 1st | CTT GGA SAC CTG MTG CGG | Moreira et al. (2007) |
| EK-1520R | SSU | Reverse | 2nd | CYG CAG GTT CAC CTA C | López-García et al. (2003) |
| 28S-1F | LSU | Forward | 2nd | ACC CGC TGA ATT TAA GCA T | Moreira et al. (2007) |
| 28S-803R | LSU | Reverse | 2nd | ACT TCG GAG GGA ACC AGC TA | Marande et al. (2009) |
| DIN464F | SSU | Forward | 3rd | TAA CAA TAC AGG GCA TCC AT | Gómez et al. (2009) |
| D1R | LSU | Forward | 1st & 2nd | ACC CGC TGA ATT TAA GCA TA | Scholin et al. (1994) |
| D3B | LSU | Reverse | 1st | TCG GAG GGA ACC AGC TAC TA | Hansen et al. (2000) |
| PF1 | SSU | Forward | 1st | GCG CTA CCT GGT TGA TCC TGC C | Hoppenrath & Leander (2010) |
| R4 | SSU | Reverse | 1st | GAT CCT TCT GCA GGT TCA CCT AC | Hoppenrath & Leander (2010) |
| SR1kuni | SSU | Forward | 1st & 2nd | CGA ATT CAA CCT GGT TGA TCC TGC CAG T | Watanabe et al. (2014) |
| SR12b | SSU | Reverse | 1st & 2nd | CGG AAAC CTT GTT ACG ACT TCT TC | Watanabe et al. (2014) |
| SR5TAK | SSU | Reverse | 2nd | ACT ACG AGC TTT TTA ACY GC | Watanabe et al. (2014) |
| SR4 | SSU | Forward | 2nd | AGG GCA AGT CTG GTG CCA G | Watanabe et al. (2014) |
| SR9p | SSU | Reverse | 2nd | AAC TAA GAA CRG CCA TGC AC | Watanabe et al. (2014) |
| SR8TAK | SSU | Forward | 2nd | GGA TTG ACA GAT TGA KAG CT | Watanabe et al. (2014) |
| SR2spin | SSU | Forward | 2nd | CAC TCA AGT TTC TGA CCT ATC | Watanabe et al. (2014) |
| SR7TAK | SSU | Reverse | 2nd | TCC WTG GCA AAT GCT TTC GC | Watanabe et al. (2014) |
| SR6 | SSU | Forward | 2nd | GTC AGA GGT GAA ATT CTT GG | Watanabe et al. (2014) |
| SR11 | SSU | Reverse | 2nd | CGC TTA CTA GGA ATT CCT CG | Watanabe et al. (2014) |
| R2 | LSU | Reverse | 1st & 2nd | ATT CGG CAG GTG AGT TGT TAC | Watanabe et al. (2014) |
| D3A | LSU | Forward | 2nd | GAC CCG TCT TGA AAC ACG GA | Watanabe et al. (2014) |
| 25R1 | LSU | Reverse | 2nd | CTT GGT CCG TGT TTC AAG AC | Watanabe et al. (2014) |
|  |  |  |  |  |  |

Gómez, F., López-García, P. & Moreira, D. (2009). Molecular phylogeny of the ocelloid-bearing dinoflagellates *Erythropsidinium* and *Warnowia* (Warnowiaceae, Dinophyceae). *J. Euk. Microb.* 56:440-5.

Hansen, G., Daugbjerg, N., & Henriksen, P. (2000). Comparative study of *Gymnodinium mikimotoi* and *Gymnodinium aureolum*, comb. nov. (= *Gyrodinium aureolum*) based on morphology, pigment composition, and molecular data. *Journal of Phycology*, 36(2), 394-410.

Hoppenrath, M. & Leander, B. S. (2010). Dinoflagellates phylogeny as inferred from heat shock protein 90 and ribosomal gene sequences. *PLoS ONE* 5: e13220.

López-García, P., Rodríguez-Valera, F., Pedrós-Alió, C., & Moreira, D. (2001). Unexpected diversity of small eukaryotes in deep-sea Antarctic plankton. *Nature*, 409(6820), 603-607.

López-García, P., Philippe, H., Gail, F., & Moreira, D. (2003). Autochthonous eukaryotic diversity in hydrothermal sediment and experimental microcolonizers at the Mid-Atlantic Ridge. *Proceedings of the National Academy of Sciences*, 100(2), 697-702.

Marande, W., López-García, P. & Moreira, D. (2009). Eukaryotic diversity and phylogeny using small- and large-subunit ribosomal RNA genes from environmental samples. *Environ. Microbiol.* 11(12):3179-88.

Moreira, D., von der Heyden, S., Bass, D., López-García, P., Chao, E. & Cavalier-Smith, T. (2007). Global eukaryote phylogeny: Combined small- and large-subunit ribosomal DNA trees support monophyly of Rhizaria, Retaria and Excavata. *Mol. Phyl. Evol.* 44:255-66.

Scholin, C.A., Herzog, M., Sogin, M. & Anderson, D.M. (1994). Identification of group- and strain-specific genetic markers for globally distributed *Alexandrium* (Dinophyceae). II. Sequence analysis of a fragment of the LSU rRNA gene. *J. Phycol.* 30:999-1011.

Watanabe, K., Miyoshi, Y., Kubo, F., Onuma, R., Murray, S., & Horiguchi, T. (2014). *Ankistrodinium armigerum* sp. nov. (Dinophyceae), a new species of heterotrophic marine sand‐dwelling dinoflagellate from Japan and Australia. *Phycological research*, 62(2), 125-135.
