# Supplementary material for: Exploration of Overlooked Aduncodinium‐Like (Pfiesteriaceae, Dinophyceae) Diversity
Source: J Eukaryot Microbiol. 2026 Aug 21;73(5):e70116. doi: 10.1111/jeu.70116 (PMC13498770; doi:10.1111/jeu.70116)
Supplement: Supplementary file 2 [file JEU-73-e70116-s002.docx]

**Table S1.** Species affiliated to the genus *Katodinium* and its current classification (according to AlgaeBase, June 2025).

**accepted** species name	basionym	synonym

**Katodinium nieuportense (type)**	Massartia nieuportensis

**Katodinium asymmetricum**	Gymnodinium asymmetricum	Massartia asymmetrica

**Katodinium auratum**

**Katodinium edax**	Massartia edax

**Katodinium galeatum**	Massartia galeata

Katodinium parvulum - uncertain taxonomic status

**Katodinium pluristigmatum**

**Katodinium redekei**	Massartia redekei

**Katodinium ruppiae**	Massartia rippiae

**Katodinium simile**

**Katodinium stigmaticum**	Gymnodinium stigmaticum

**Katodinium thiophilum**	Massartia thiophilum

**Katodinium tubulatum**	Massartia tubulata

**Katodinium uncinatum**	Massartia uncinate

**Katodinium viride**

***Aduncodinium glandula***	Gymnodinium glandula	Katodinium glandula

**Borghiella pascheri**	Katodinium hiemale

Massartia hiemale (basionym)

**Gymnodinium astigmatum**	Katodinium astigmatum nom. inval.

**Gymnodinium bohemicum**	Gymnodinium bohemicum	Katodinium bohemicum

**Gymnodinium dorsalisulcum**	Katodinium dorsalisulcum

**Gymnodinium fontinale**	Katodinium fontinale

**Gymnodinium hyperxanthoides**	Massartia hyperxanthoides	Katodinium hyperxanthoides

**Gymnodinium hyperxanthum**	Massartia hyperxanthum	Katodinium hyperxanthum

**Gymnodinium intermedium**	Katodinium intermedium nom. inval.

**Gymnodinium mazuricum**	Katodinium mazuricum

**Gymnodinium partitum**	Katodinium monadicum

Glenodin. monadicum (basionym)

Katodinium polyplastidum

**Gymnodinium palustre***	Katodinium palustre

**Gymnodinium planum**	Massartia plana	Katodinium planum

**Gymnodinium pratense**	Massartia pratensis	Katodinium pratense

**Gymnodinium sanctipaulense**	Katodinium sanctipaulense

**Gymnodinium schilleri**	Massartia schilleri	Katodinium schilleri

**Gymnodinium spirodinioides**	Katodinium spirodinioides

**Gymnodinium vernum**	Katodinium vernale

**Gymnodinium vierae**	Katodinium vierae

**Gyrodinium molopicum**	Massartia molopicum	Katodinium molopicum

**Heterocapsa rotundata**	*Amphidinium* rotundatum	Katodinium rotundatum

Katodinium minutum

**Kapelodinium vestifici**	Katodinium glaucum

Lebouridinium glaucum

Spirodinium glaucum (basionym)

**Nusuttodinium crassifilum**	Massartia crassifilum	Katodinium crassifilum

**Nusuttodinium montanum**	Massartia montanum	Katodinium montanum

**Opisthoaulax campylops**	Massartia campylops	Katodinium campylops

**Opisthoaulax fastigata**	Katodinium fastigatum

**Opisthoaulax musei**	Gymnodinium musaei	Katodinium musaei

**Opisthoaulax woloszynskae**	Massartia woloszynskae	Katodinium woloszynskae

Katodinium notatum

**Opisthoaulax piscinalis**	Katodinium piscinale

**Opisthoaulax tetragonops**	Massartia tetragonops	Katodinium tetragonops

Katodinium ptyrticum

Massartia ptyrticum (basionym)

**Opisthoaulax vorticella**	Gymnodinium vorticella	Katodinium vorticella

**Speroidium austriacum**	Massartia austriaca	Katodinium austriacum

**Speroidium fungiforme**	Gymnodinium fungiforme	Katodinium fungiforme

* Kretschmann et al. (2015, Protist 166: 621-637) transferred the species into the genus *Spiniferodinium*, but Moestrup & Calado (2018, Süßwasserflora von Mitteleuropa, Springer Spektrum) regarded *S. palustre* as nomenclatural synonym.
