# Supplementary material for: Exploration of Overlooked Aduncodinium‐Like (Pfiesteriaceae, Dinophyceae) Diversity
Source: J Eukaryot Microbiol. 2026 Aug 21;73(5):e70116. doi: 10.1111/jeu.70116 (PMC13498770; doi:10.1111/jeu.70116)
Supplement: Supplementary file 3 [file JEU-73-e70116-s003.docx]

**Figure S1.** Light microscopy images of *Aduncodinium* cf. *robustum* from A-B) Castelldefels, Spain, and C-F) Kuwait (Al-Yamani & Saburova, 2010). Arrowheads indicate the apical hook. The arrow indicates the left sulcal list. n: nucleus; p: pusule. Scale bars = 10 µm.
